# Supplementary material for: Human lipoproteins comprise at least 12 different classes that are lognormally distributed
Source: PLoS One. 2022 Nov 10;17(11):e0275066. doi: 10.1371/journal.pone.0275066 (PMC9648703; doi:10.1371/journal.pone.0275066)
Supplement: S1 File — (ZIP) [file pone.0275066.s001.zip › supporting/fig/Ch/Ch.htm]

Ch


## Supporting Information

  

Click to enlarge

### Cholesterol

| CM1 | CM2 | VLDL | Lp(a) |
| --- | --- | --- | --- |
|  |  |  |  |
| Tg | LDL1 | LAC1 | LDL2 |
|  |  |  |  |
| LAC2 | mHDL | HDL1 | HDL2 |
|  |  |  |  |

  

S6 Fig. Normal QQ plot of logarithms of raw data. Cholesterol.   
The first order regression lines were drawn by using the robust estimators: trimmed mean and MAD.  
Those parameters were also used for the normalizaion of data (integrated and applied for the  QQ plots )  
and the estimations of the 95% interval of  the standard values.

  
  

back to the home

  
