# Supplementary material for: Human lipoproteins comprise at least 12 different classes that are lognormally distributed
Source: PLoS One. 2022 Nov 10;17(11):e0275066. doi: 10.1371/journal.pone.0275066 (PMC9648703; doi:10.1371/journal.pone.0275066)
Supplement: S1 File — (ZIP) [file pone.0275066.s001.zip › supporting/fig/withoutTG/without.htm]

TG


## Supporting Information

  

Click to enlarge

### TG

| With TR | Without TR |
| --- | --- |
|  |  |
| With TR | Without TR |
|  |  |
| With TR | Without TR |
|  |  |

  
  
  

back to the home

  
