# Supplementary material for: Human lipoproteins comprise at least 12 different classes that are lognormally distributed
Source: PLoS One. 2022 Nov 10;17(11):e0275066. doi: 10.1371/journal.pone.0275066 (PMC9648703; doi:10.1371/journal.pone.0275066)
Supplement: S1 File — (ZIP) [file pone.0275066.s001.zip › supporting/index.htm]

Support


# Supporting Information

for "Human lipoproteins comprise at least 12 different classes that are lognormally distributed."  
Konishi et al. 2021

Click images to enlarge

### S1 Fig.

### S2 Fig.

### S3 Fig.

### S4 Fig.

### S5 Fig.

### S6 Fig.

### S7 Fig.

### S8 Fig.

## Appendix: The normal and lognormal distributions

The level of lipids in the serum was monitored and adjusted to an appropriate amount. Possible factors affecting the level of lipids were simulated using Dice.

### S9 Fig.

### S10 Fig.

## Tables

### S1 Table.

### S2 Table.

## Data
