# Supplementary material for: Human lipoproteins comprise at least 12 different classes that are lognormally distributed
Source: PLoS One. 2022 Nov 10;17(11):e0275066. doi: 10.1371/journal.pone.0275066 (PMC9648703; doi:10.1371/journal.pone.0275066)
Supplement: S1 File — (ZIP) [file pone.0275066.s001.zip › supporting/pages/S10Fig.htm]

S10


### S10 Fig.

| A | B |
| --- | --- |
|  |  |

Fig. S10 Histograms of the simulated levels. A. additive and B. multiplicative models.

When factors act additively, lipid levels are normally distributed (**A**). This is because the sum of random numbers will take a normal distribution (central limit theorem). The 95% range is represented by a dotted line, and the median is represented by a straight line. The median is at the centre of the distribution, and the upper and lower bounds are symmetric.
However, if the factors behave multiplicatively, the distribution is lognormal (**B**). The distribution was highly skewed. The upper and lower limits are not symmetrical.

  
  
R codes
  

back to the home
