# Supplementary material for: Human lipoproteins comprise at least 12 different classes that are lognormally distributed
Source: PLoS One. 2022 Nov 10;17(11):e0275066. doi: 10.1371/journal.pone.0275066 (PMC9648703; doi:10.1371/journal.pone.0275066)
Supplement: S1 File — (ZIP) [file pone.0275066.s001.zip › supporting/pages/S1Fig.htm]

S1


### S1 Fig.

|  |
| --- |
|  |

Fig. S1 The age of volunteers.

  

back to the home
