# Supplementary material for: Human lipoproteins comprise at least 12 different classes that are lognormally distributed
Source: PLoS One. 2022 Nov 10;17(11):e0275066. doi: 10.1371/journal.pone.0275066 (PMC9648703; doi:10.1371/journal.pone.0275066)
Supplement: S1 File — (ZIP) [file pone.0275066.s001.zip › supporting/pages/S1Table.htm]

S1T


## S1 Table

### Position and scale(nm)

|  |  |  |  |  |  |  |  |  |  |  |  |  |
| --- | --- | --- | --- | --- | --- | --- | --- | --- | --- | --- | --- | --- |
|  | CM1 | CM2 | VLDL | Lp(a) | TR | LDL1 | LAC1 | LDL2 | LAC2 | mHDL | HDL1 | HDL2 |
| ƒÊ (min) | 17.7 | 19.8 | 20.9 | 21.8 | 23.0 | 23.6 | 25.0 | 26.5 | 27.0 | 27.7 | 28.5 | 29.6 |
| ƒÐ (min) | 0.7 | 0.9 | 0.7 | 0.5 | 0.7 | 0.6 | 0.4 | 0.5 | 0.5 | 0.5 | 0.6 | 0.6 |

  
  

### Diameter (nm) at the scale ƒÊ

|  |  |  |  |  |  |  |  |  |  |  |  |  |
| --- | --- | --- | --- | --- | --- | --- | --- | --- | --- | --- | --- | --- |
|  | CM1 | CM2 | VLDL | Lp(a) | TR | LDL1 | LAC1 | LDL2 | LAC2 | mHDL | HDL1 | HDL2 |
| upper (nm) | 98.9 | 63.8 | 46.4 | 35.5 | 27.1 | 22.6 | 16.4 | 10.8 | 9.6 | 8.0 | 6.7 | 5.0 |
| mean (nm) | 94.3 | 56.3 | 42.8 | 34.5 | 25.6 | 22.0 | 15.5 | 10.7 | 9.5 | 7.9 | 6.5 | 4.9 |
| lower (nm) | 90.0 | 49.6 | 39.5 | 33.5 | 24.2 | 21.5 | 14.7 | 10.5 | 9.4 | 7.8 | 6.3 | 4.8 |

S1 Tables   
The standard values and 95% intervals.

  

back to the home
