# Supplementary material for: Human lipoproteins comprise at least 12 different classes that are lognormally distributed
Source: PLoS One. 2022 Nov 10;17(11):e0275066. doi: 10.1371/journal.pone.0275066 (PMC9648703; doi:10.1371/journal.pone.0275066)
Supplement: S1 File — (ZIP) [file pone.0275066.s001.zip › supporting/pages/S2Fig.htm]

S2


### S2 Fig.

|  |  |
| --- | --- |
|  |  |

Fig. S2 The need for class TG-rich class (TR).   
This class was fairly minor and was lacking in rats.   
It is unique in that it is smaller and very low in cholesterol.   
However, fitting is difficult without this.   
The fitting of triacylglycerol is shown.

  
  

back to the home
