# Supplementary material for: Human lipoproteins comprise at least 12 different classes that are lognormally distributed
Source: PLoS One. 2022 Nov 10;17(11):e0275066. doi: 10.1371/journal.pone.0275066 (PMC9648703; doi:10.1371/journal.pone.0275066)
Supplement: S1 File — (ZIP) [file pone.0275066.s001.zip › supporting/pages/S2Table.htm]

S2T


## S2 Table

### Cholesterol (mg/dL)

|  | CM1 | CM2 | VLDL | LDL1 | Lp(a) | TR | LAC1 | LDL2 | LAC2 | mHDL | HDL1 | HDL2 |
| --- | --- | --- | --- | --- | --- | --- | --- | --- | --- | --- | --- | --- |
| upper | 5.8 | 21.9 | 21.8 | 158.5 | 34.3 | 0.0 | 4.5 | 87.6 | 51.5 | 5.7 | 1.7 | 0.7 |
| mean | 0.4 | 1.5 | 4.7 | 98.3 | 14.7 | 0.0 | 2.8 | 23.7 | 35.6 | 2.9 | 1.4 | 0.5 |
| lower | 0.0 | 0.1 | 1.0 | 61.0 | 6.3 | 0.0 | 1.7 | 6.4 | 24.6 | 1.5 | 1.2 | 0.3 |

  
  

### TG (mg/dL)

|  | CM1 | CM2 | VLDL | Lp(a) | TR | LDL1 | LAC1 | LDL2 | LAC2 | mHDL | HDL1 | HDL2 |
| --- | --- | --- | --- | --- | --- | --- | --- | --- | --- | --- | --- | --- |
| upper | 49.5 | 127.2 | 112.7 | 97.4 | 25.3 | 18.8 | 1.7 | 13.7 | 14.1 | 2.0 | 1.4 | 1.1 |
| mean | 2.0 | 7.1 | 15.4 | 10.9 | 14.1 | 10.9 | 0.8 | 6.0 | 5.0 | 0.4 | 0.9 | 0.2 |
| lower | 0.1 | 0.4 | 2.1 | 1.2 | 7.9 | 6.3 | 0.4 | 2.6 | 1.8 | 0.1 | 0.6 | 0.0 |

S2 Table   
The standard values and 95% intervals.

  
  
  

back to the home
