# Supplementary material for: Human lipoproteins comprise at least 12 different classes that are lognormally distributed
Source: PLoS One. 2022 Nov 10;17(11):e0275066. doi: 10.1371/journal.pone.0275066 (PMC9648703; doi:10.1371/journal.pone.0275066)
Supplement: S1 File — (ZIP) [file pone.0275066.s001.zip › supporting/pages/S4Fig.htm]

S4


### S4 Fig.

| A | B | C |
| --- | --- | --- |
|  |  |  |

Fig. S4 Another version of Fig. 2 using a different sample.   
**A.** ApoB, **B.** LACs, **C.** ApoA.

  
  

back to the home
