# Supplementary material for: Human lipoproteins comprise at least 12 different classes that are lognormally distributed
Source: PLoS One. 2022 Nov 10;17(11):e0275066. doi: 10.1371/journal.pone.0275066 (PMC9648703; doi:10.1371/journal.pone.0275066)
Supplement: S1 File — (ZIP) [file pone.0275066.s001.zip › supporting/pages/S5Fig.htm]

S5


### S5 Fig.

| A | B | C |
| --- | --- | --- |
|  |  |  |
| |  |  | | --- | --- | | Pearson's r | p-value | | 0.1144 | 0.4435 | | |  |  | | --- | --- | | Pearson's r | p-value | | -0.0606 | 0.6854 | | |  |  | | --- | --- | | Pearson's r | p-value | | -0.2227 | 0.1323 | |
|  |
| D | E | F |
|  |  |  |
| |  |  | | --- | --- | | Pearson's r | p-value | | 0.5832 | 1.692e-05 | | |  |  | | --- | --- | | Pearson's r | p-value | | 0.9061 | < 2.2e-16 | | |  |  | | --- | --- | | Pearson's r | p-value | | 0.1065 | 0.4759 | |

Fig. S5 Correlation between classes.

  
  

back to the home
