# Supplementary material for: Human lipoproteins comprise at least 12 different classes that are lognormally distributed
Source: PLoS One. 2022 Nov 10;17(11):e0275066. doi: 10.1371/journal.pone.0275066 (PMC9648703; doi:10.1371/journal.pone.0275066)
Supplement: S1 File — (ZIP) [file pone.0275066.s001.zip › supporting/pages/S7Fig.htm]

S7


### S7 Fig.

|  |
| --- |
|  |

Fig. S7 A part of SDS-PAGE, fractions 15-32, with the elution pattern superimposed.   
Immunoglobulins are marked. IgM formed a large molecule of ca 20 nm; the size was comparable to LDL1.  
Also, IgG was 8 nm; some of those further bound to complement factor C3 as well as H, and the size became comparable with LDL2.

  

back to the home
