# Supplementary material for: Human lipoproteins comprise at least 12 different classes that are lognormally distributed
Source: PLoS One. 2022 Nov 10;17(11):e0275066. doi: 10.1371/journal.pone.0275066 (PMC9648703; doi:10.1371/journal.pone.0275066)
Supplement: S1 File — (ZIP) [file pone.0275066.s001.zip › supporting/pages/S9Fig.htm]

S9


### S9 Fig.

| A | B |
| --- | --- |
|  |  |

Fig. S9 Sums of randomly selected five samples.

Five numbers were randomly sampled from each of these two distributions, and the sum was obtained. In the additive model, the sum of the samples converges to a fixed point (**A**). The flicker of this value was small. Therefore, this sum is a good indicator of these levels. However, in the multiplicative model, the distribution of sums is biased (**B**). Owing to the chance of sampling, the obtained value changes significantly. According to the central limit theorem, if the number of samples is sufficient, the sum should have a normal distribution. In fact, the skewness of the distribution was less than that of the original distribution (Fig. S11B).   
However, the lognormality feature remained unchanged (**B**). Therefore, the sum of the samples is not informative in the multiplicative model. Theoretically, the geometric mean is appropriate; however, this cannot be estimated experimentally. The levels of LDL and HDL measured by the conventional enzymatic method are not randomly selected; however, in the sense of instability, they will have a similar weakness.  
Additionally, note that the interval ranges inevitably become asymmetric to the standards: the higher is always wider.

  

back to the home
