# Supplementary figures and images for: Human lipoproteins comprise at least 12 different classes that are lognormally distributed
Source: PLoS One. 2022 Nov 10;17(11):e0275066. doi: 10.1371/journal.pone.0275066 (PMC9648703; doi:10.1371/journal.pone.0275066)

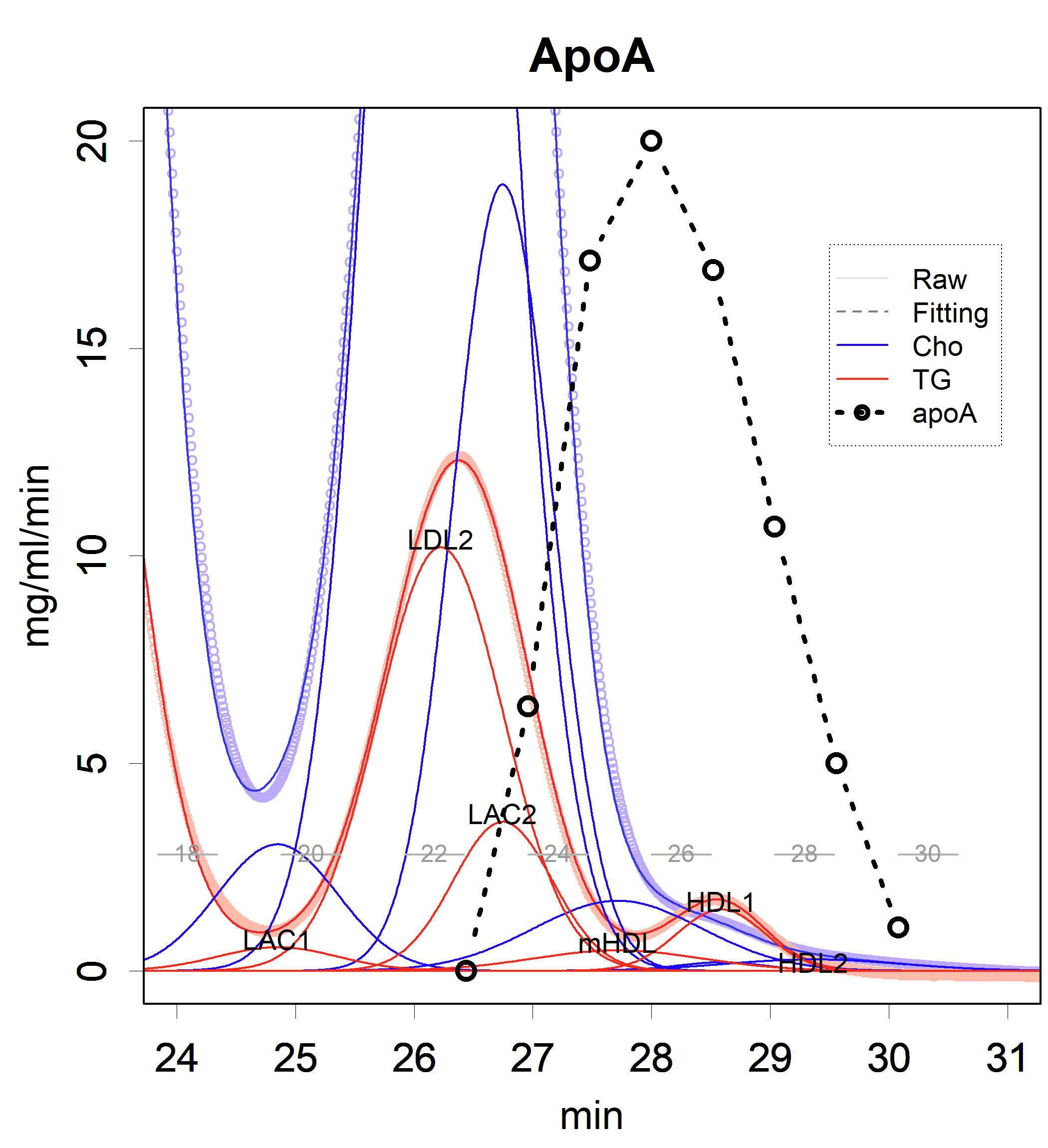

Supplement: S1 File — (ZIP) [file pone.0275066.s001.zip › supporting/fig/apoA_401.png]

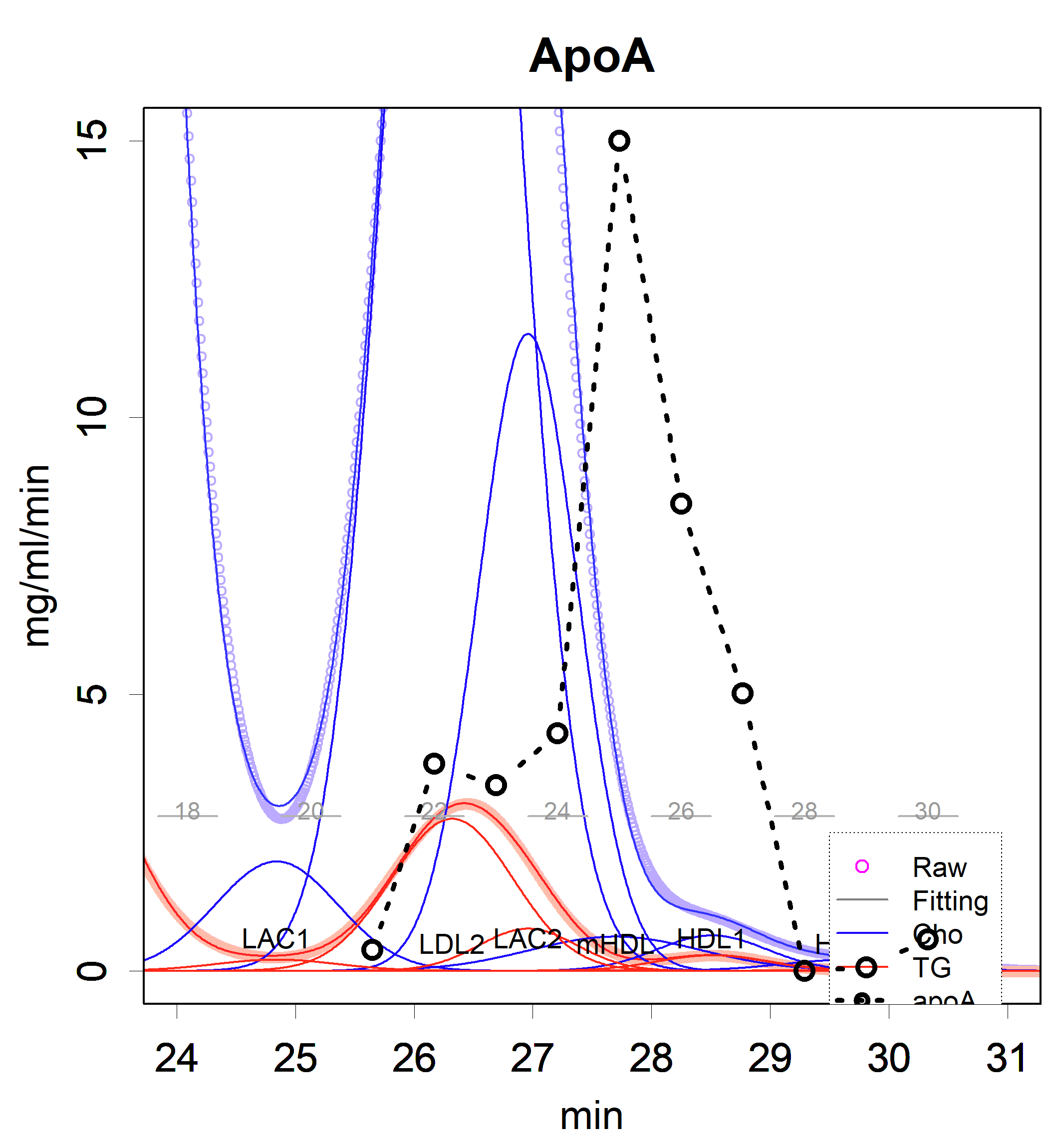

Supplement: S1 File — (ZIP) [file pone.0275066.s001.zip › supporting/fig/apoA_402.png]

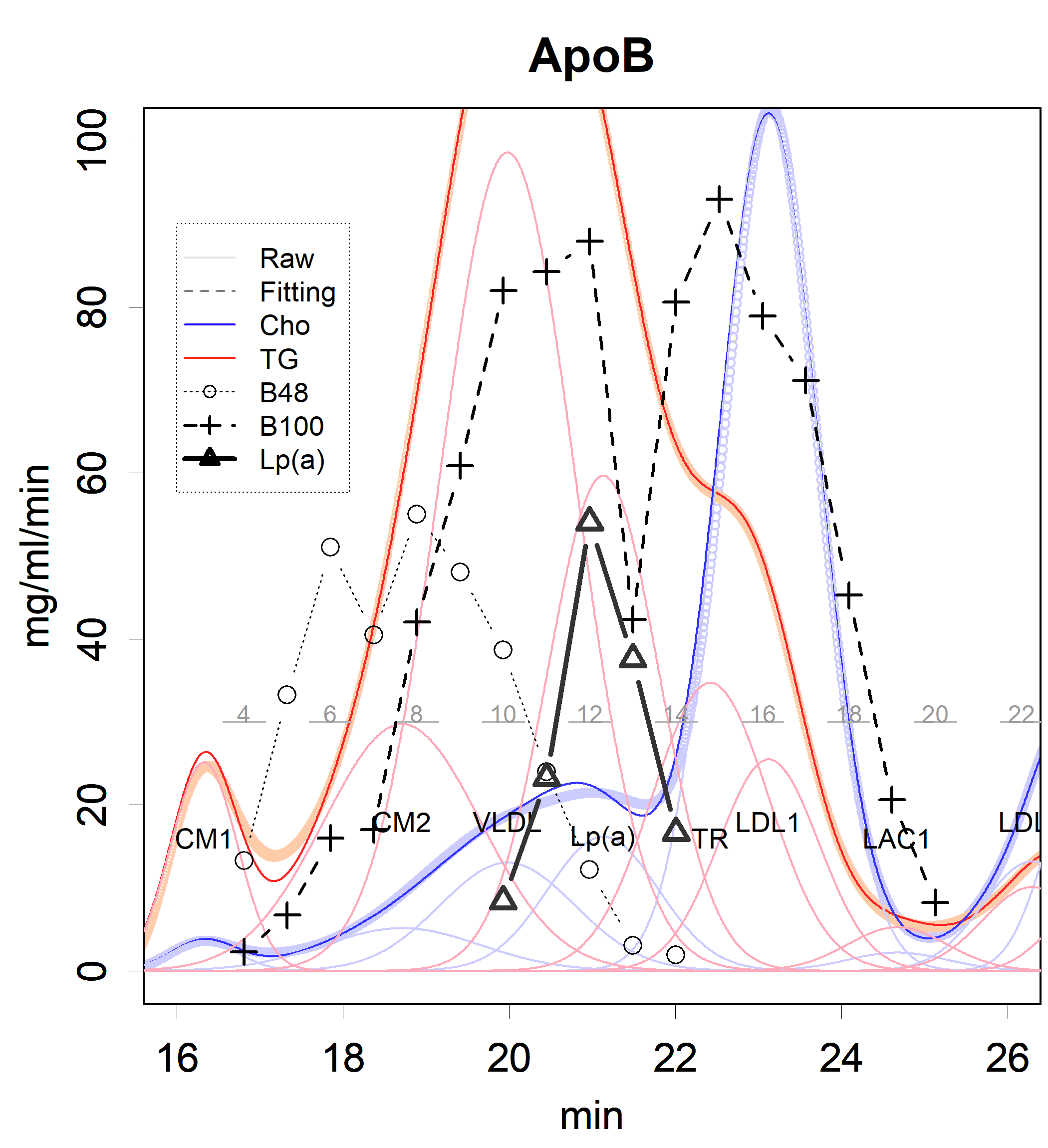

Supplement: S1 File — (ZIP) [file pone.0275066.s001.zip › supporting/fig/apoB_20.png]

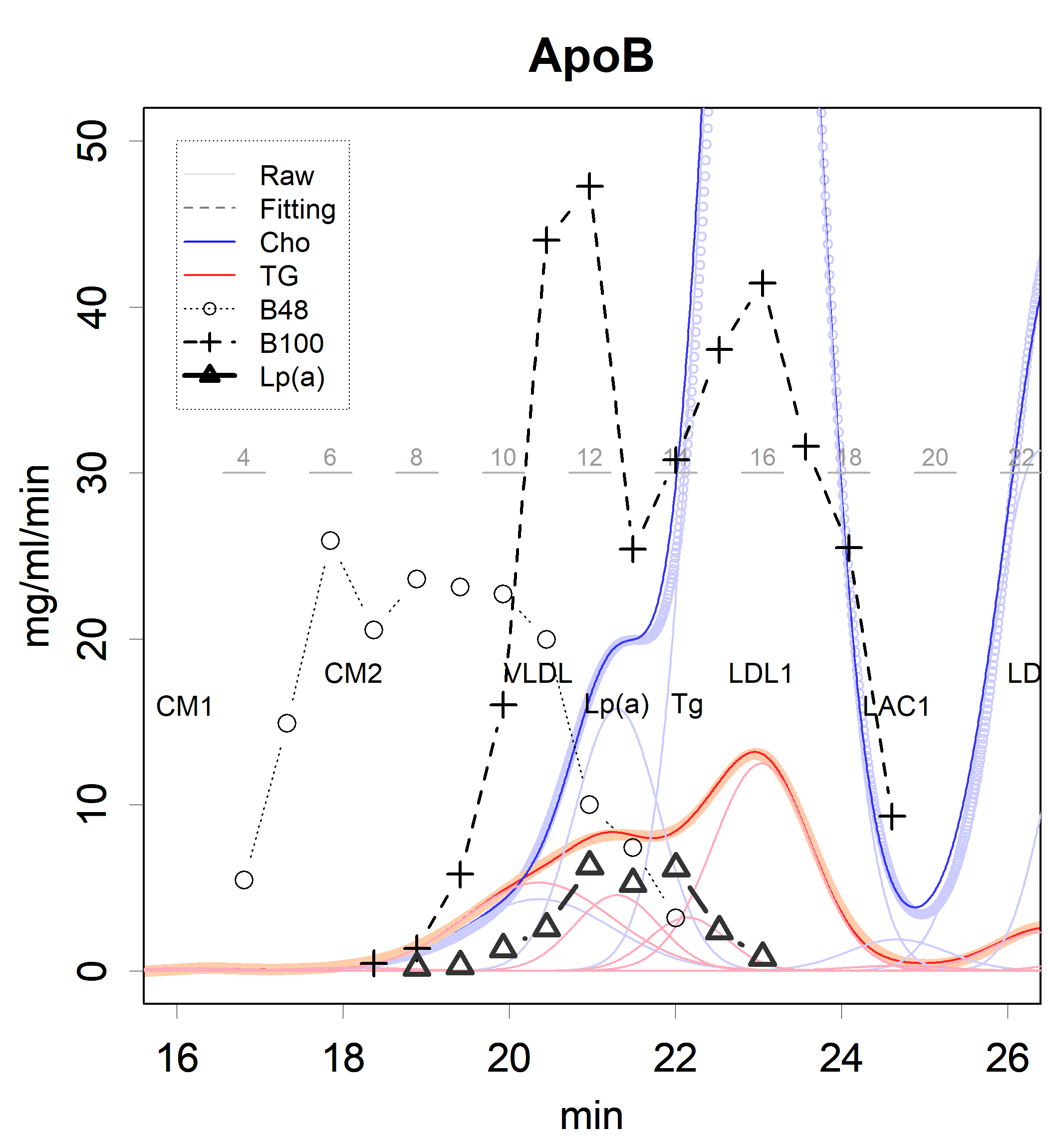

Supplement: S1 File — (ZIP) [file pone.0275066.s001.zip › supporting/fig/apoB_37.png]

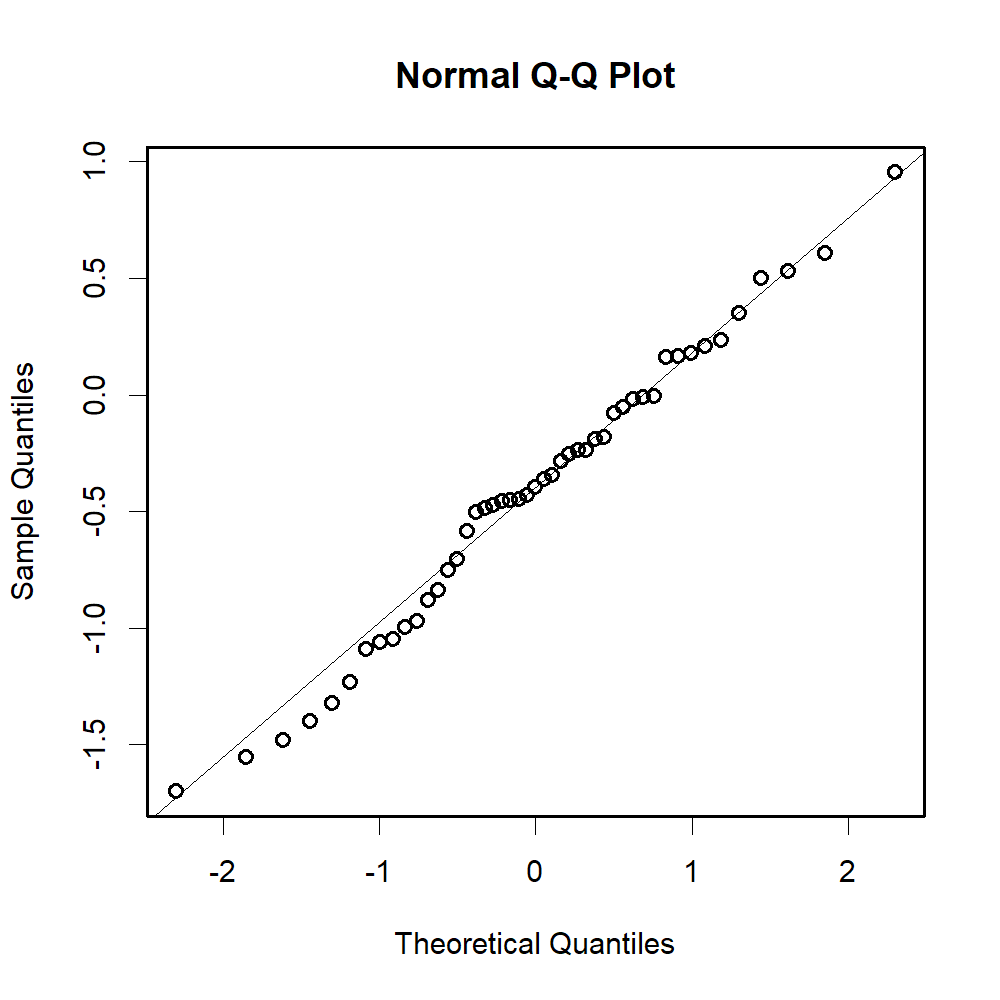

Supplement: S1 File — (ZIP) [file pone.0275066.s001.zip › supporting/fig/Ch/CM1.png]

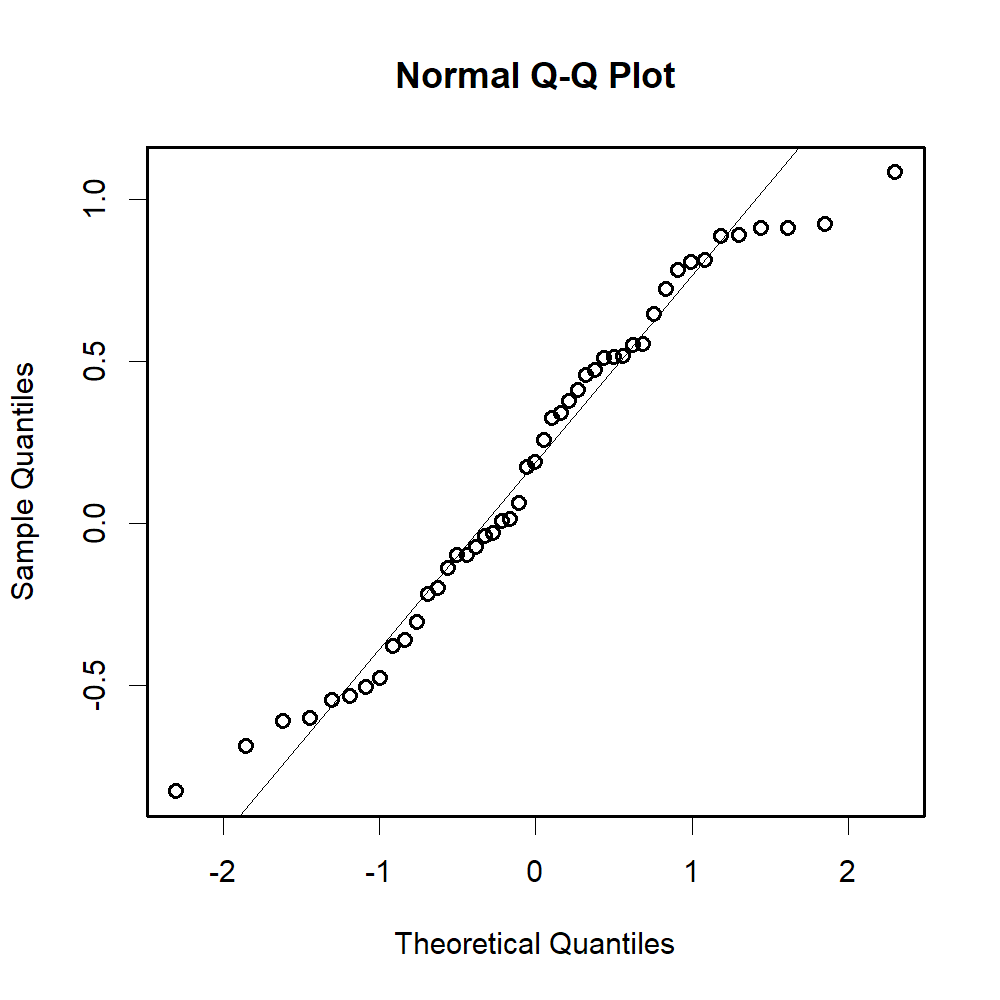

Supplement: S1 File — (ZIP) [file pone.0275066.s001.zip › supporting/fig/Ch/CM2.png]

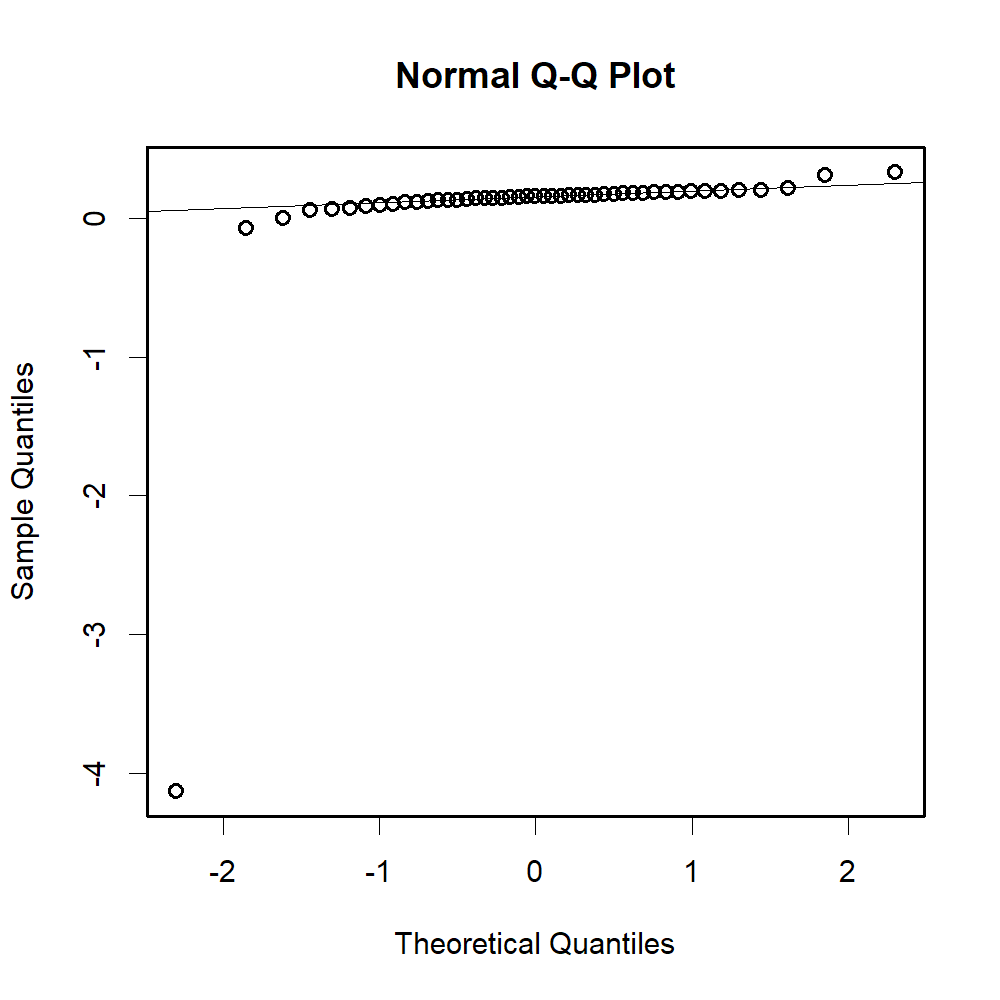

Supplement: S1 File — (ZIP) [file pone.0275066.s001.zip › supporting/fig/Ch/HDL1.png]

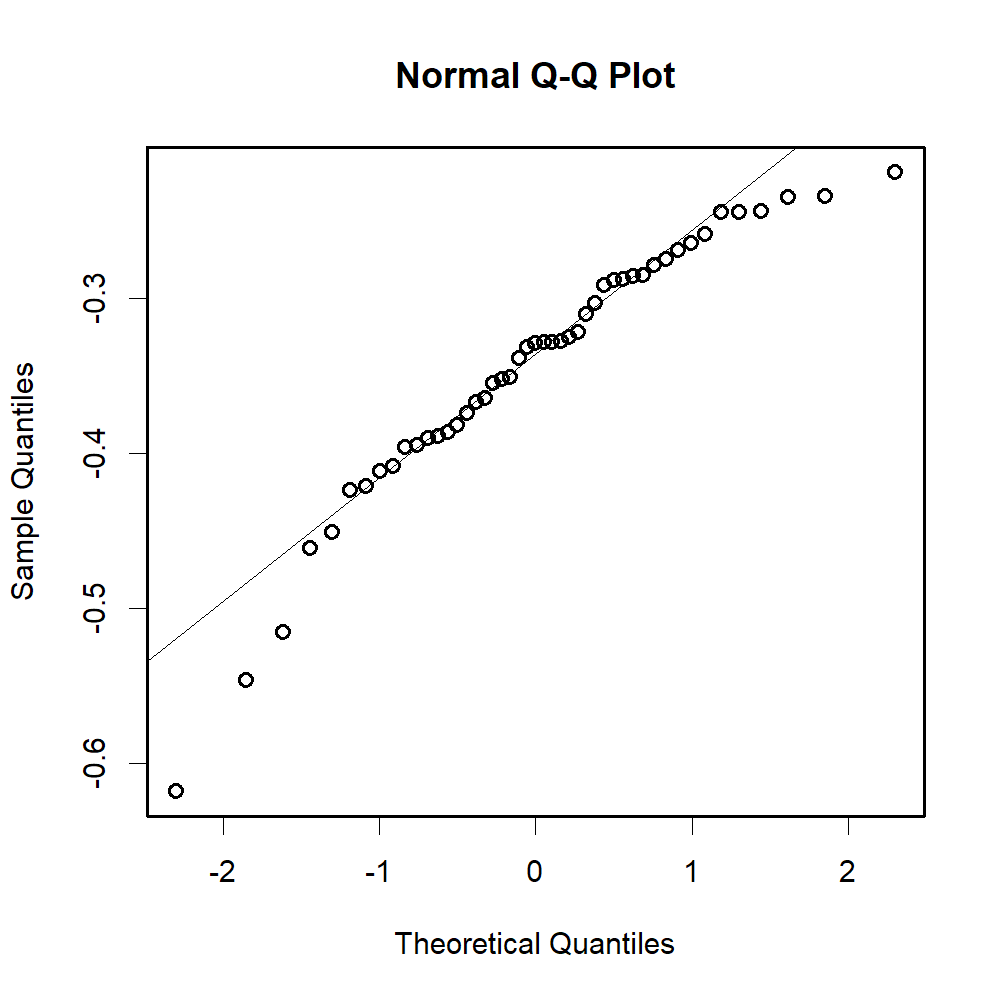

Supplement: S1 File — (ZIP) [file pone.0275066.s001.zip › supporting/fig/Ch/HDL2.png]

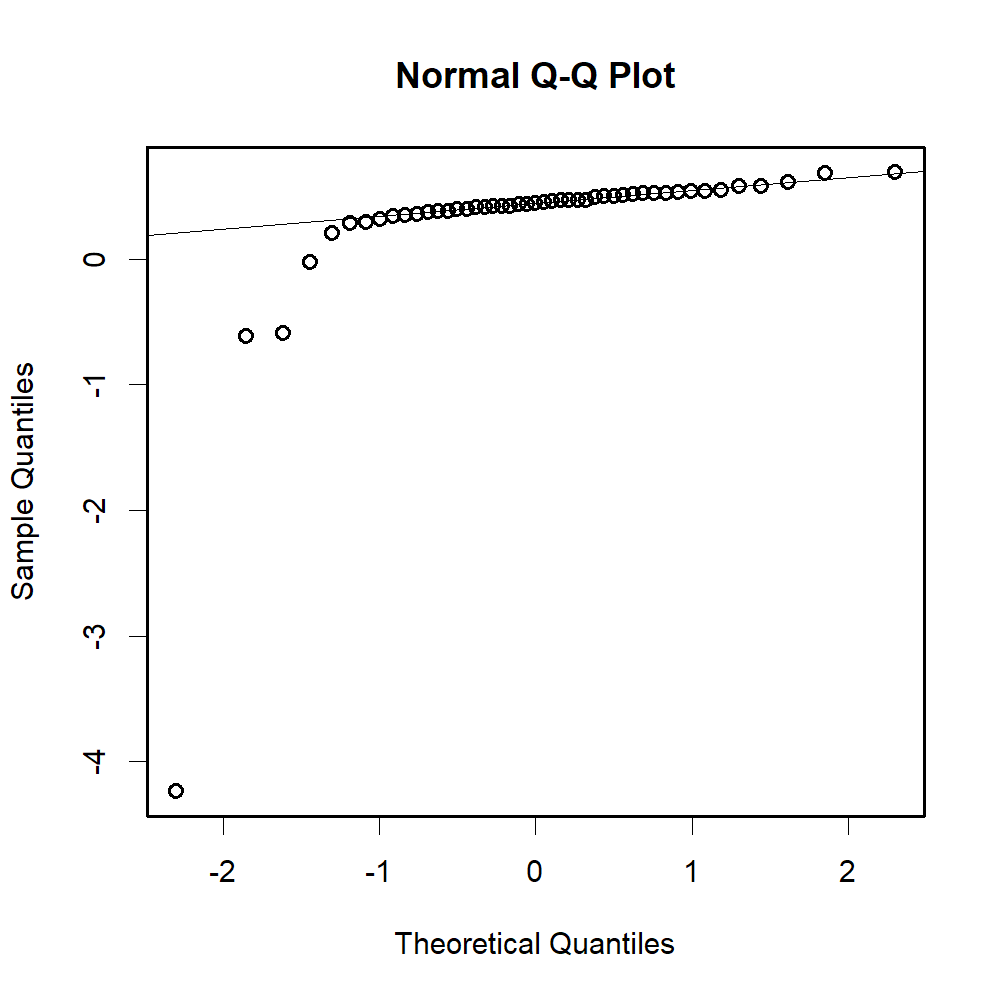

Supplement: S1 File — (ZIP) [file pone.0275066.s001.zip › supporting/fig/Ch/LAC1.png]

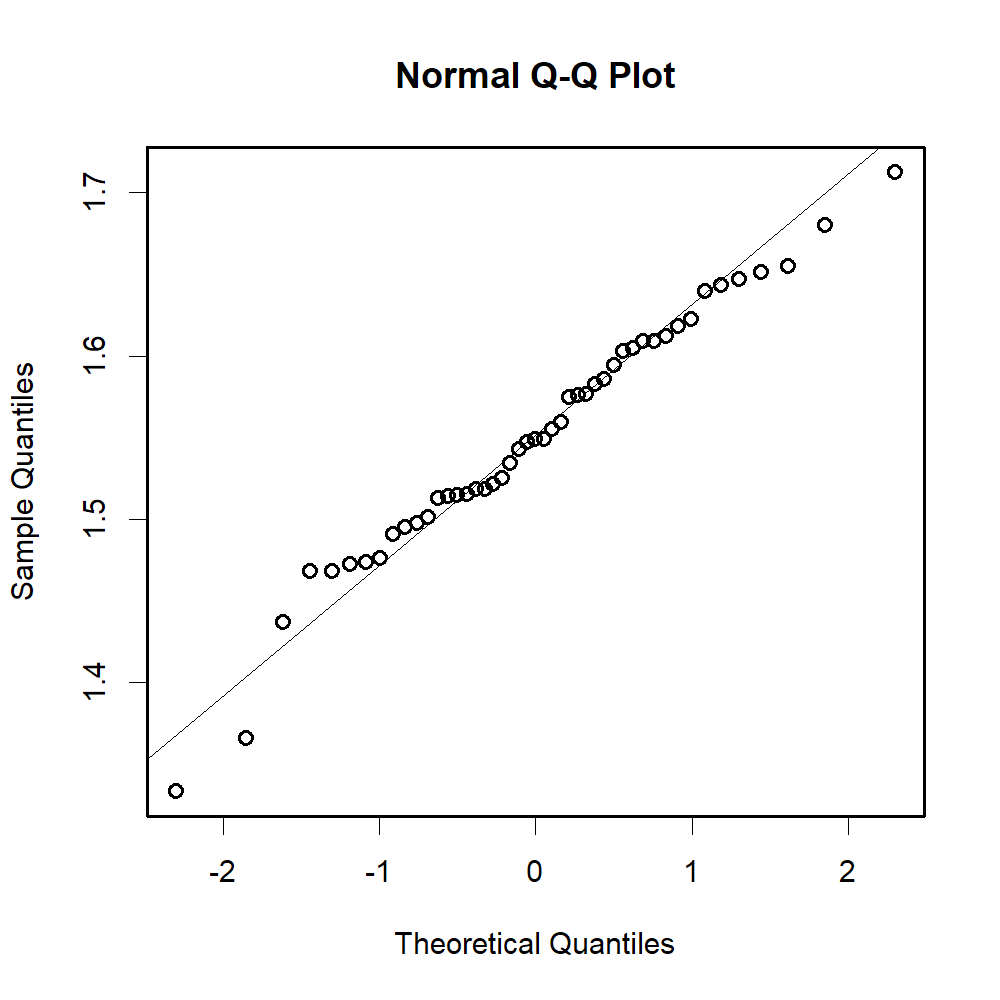

Supplement: S1 File — (ZIP) [file pone.0275066.s001.zip › supporting/fig/Ch/LAC2.png]

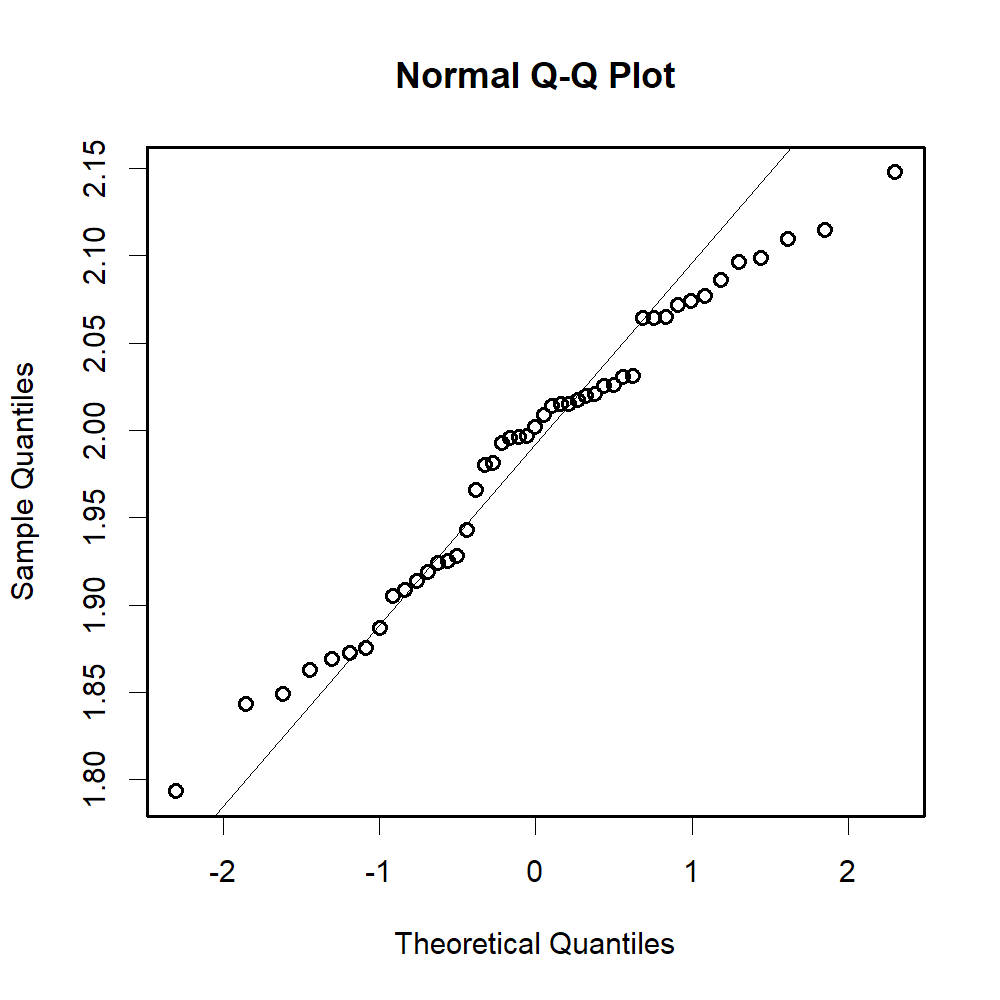

Supplement: S1 File — (ZIP) [file pone.0275066.s001.zip › supporting/fig/Ch/LDL1.png]

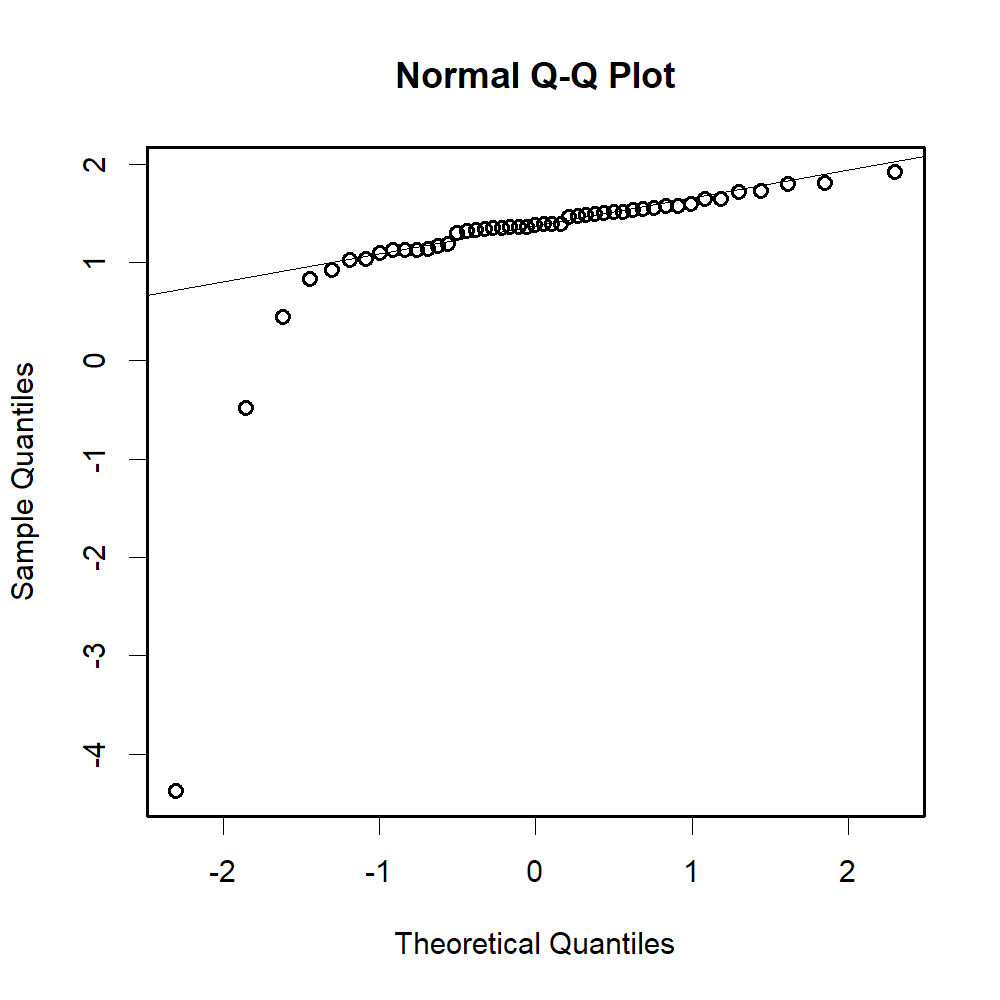

Supplement: S1 File — (ZIP) [file pone.0275066.s001.zip › supporting/fig/Ch/LDL2.png]

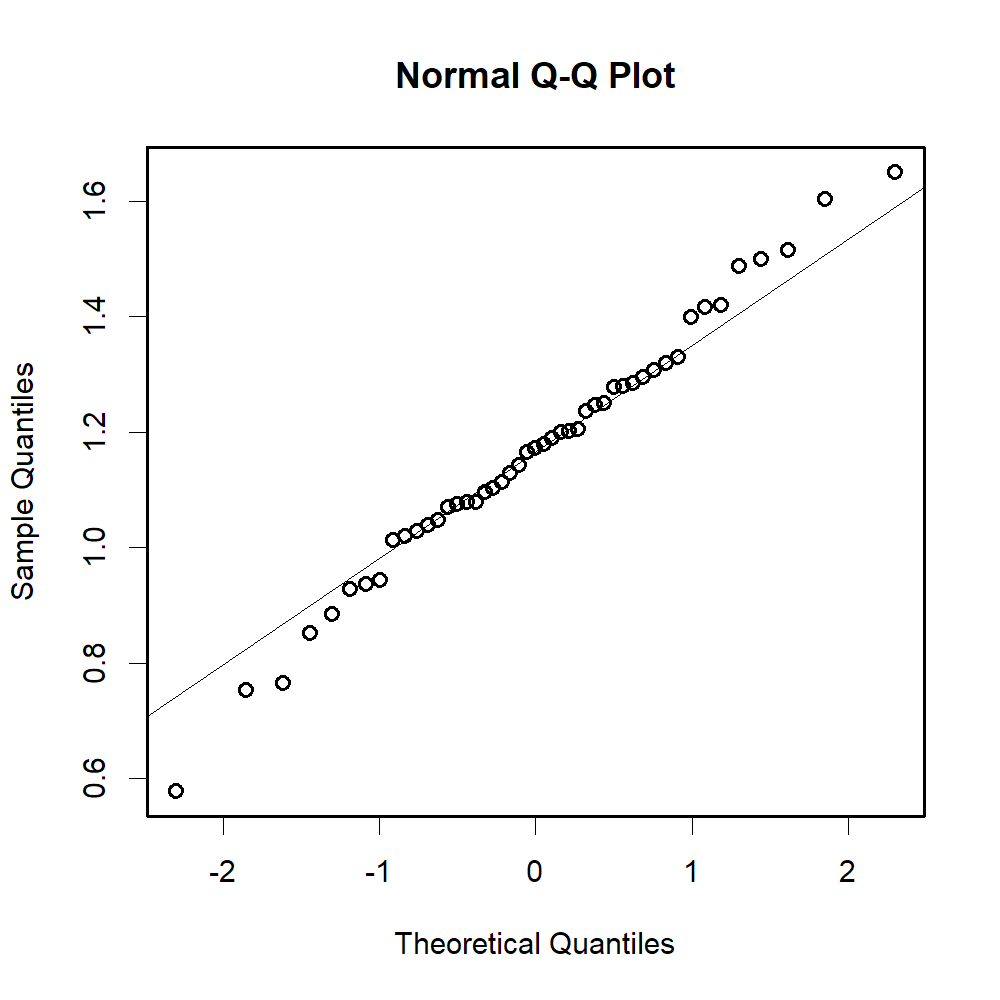

Supplement: S1 File — (ZIP) [file pone.0275066.s001.zip › supporting/fig/Ch/Lp(a).png]

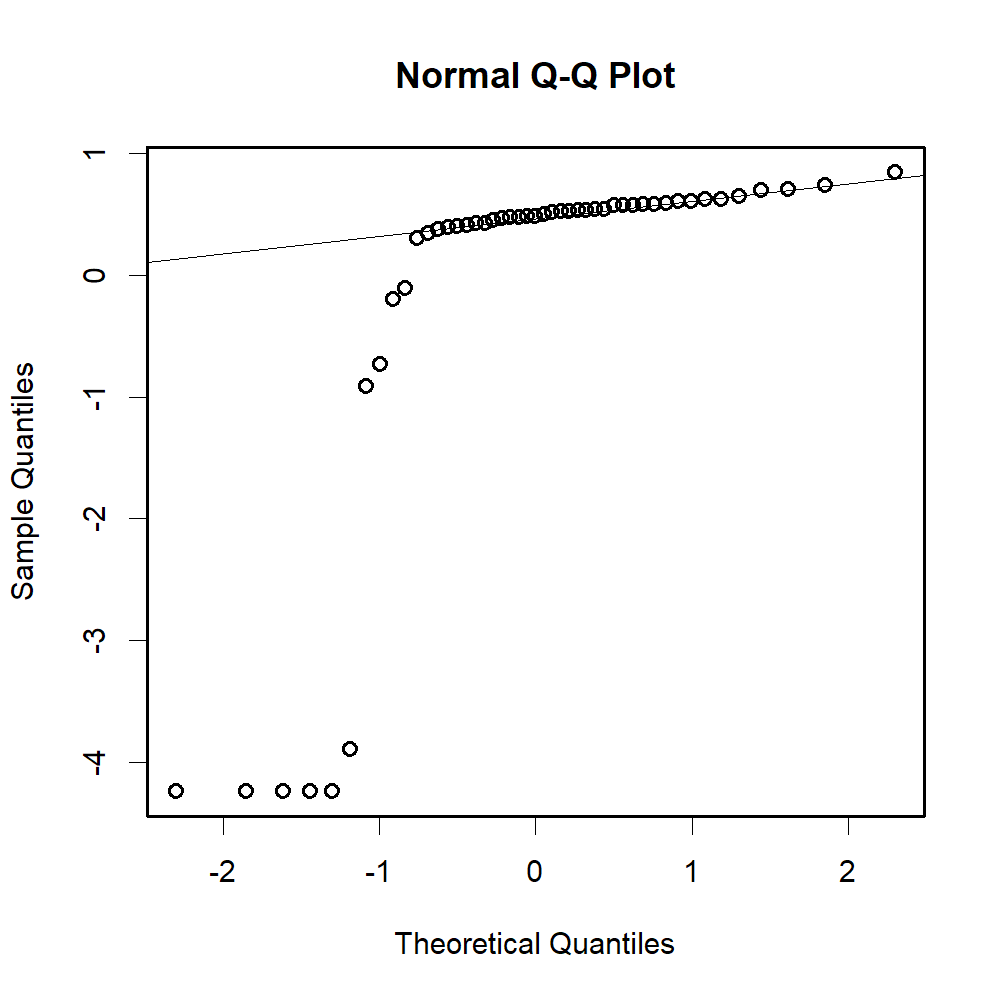

Supplement: S1 File — (ZIP) [file pone.0275066.s001.zip › supporting/fig/Ch/mHDL.png]

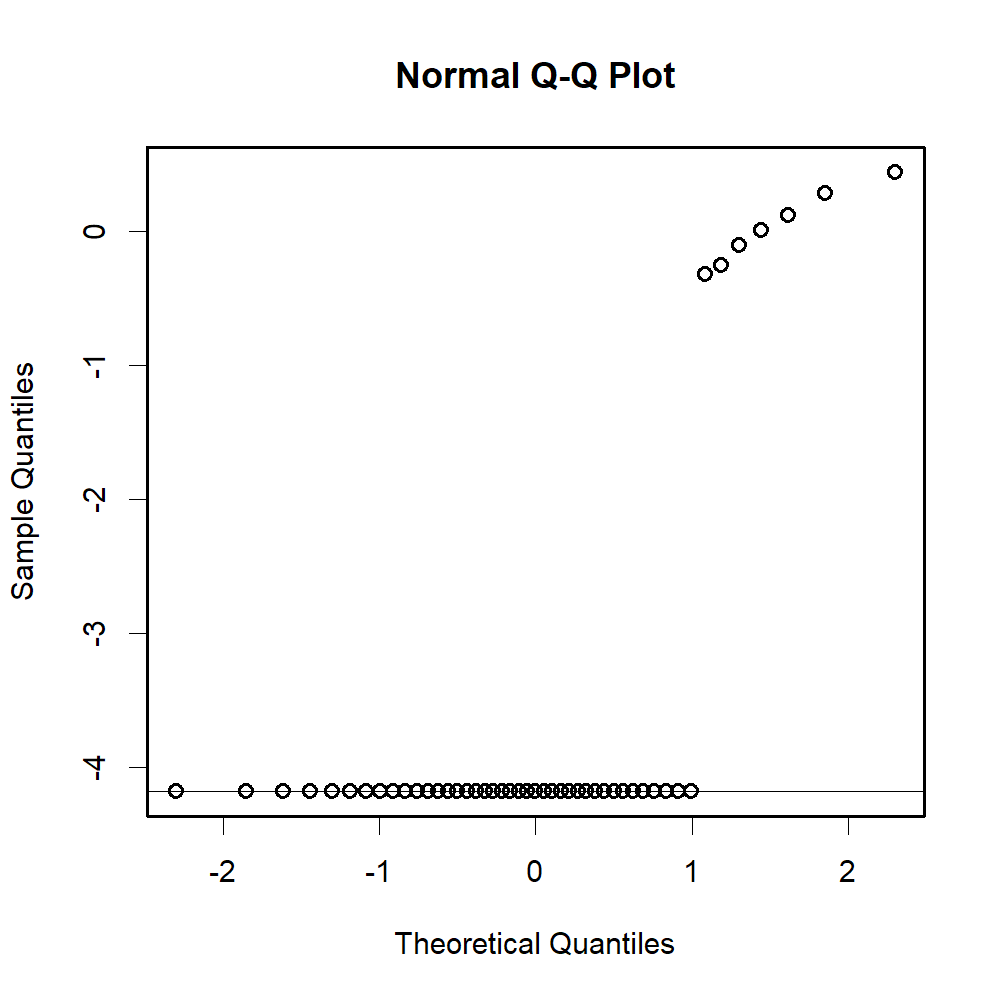

Supplement: S1 File — (ZIP) [file pone.0275066.s001.zip › supporting/fig/Ch/Tg.png]

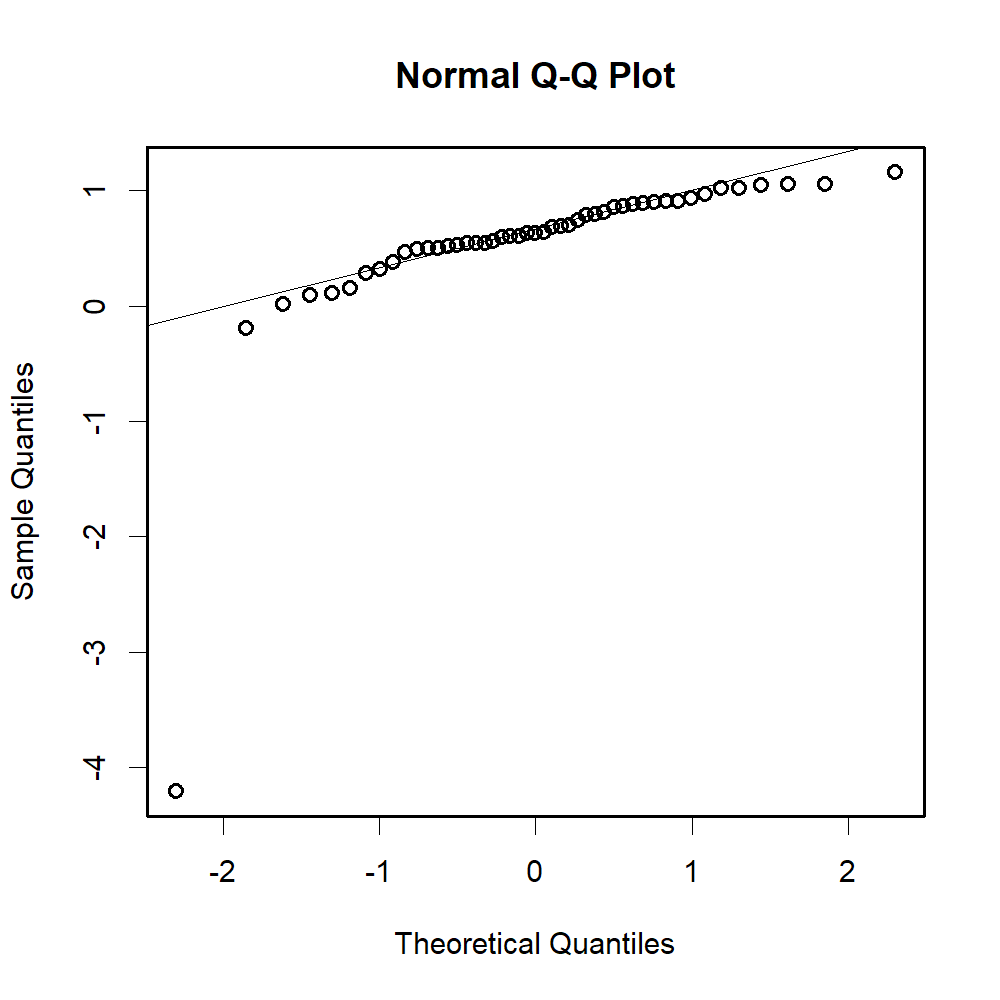

Supplement: S1 File — (ZIP) [file pone.0275066.s001.zip › supporting/fig/Ch/VLDL.png]

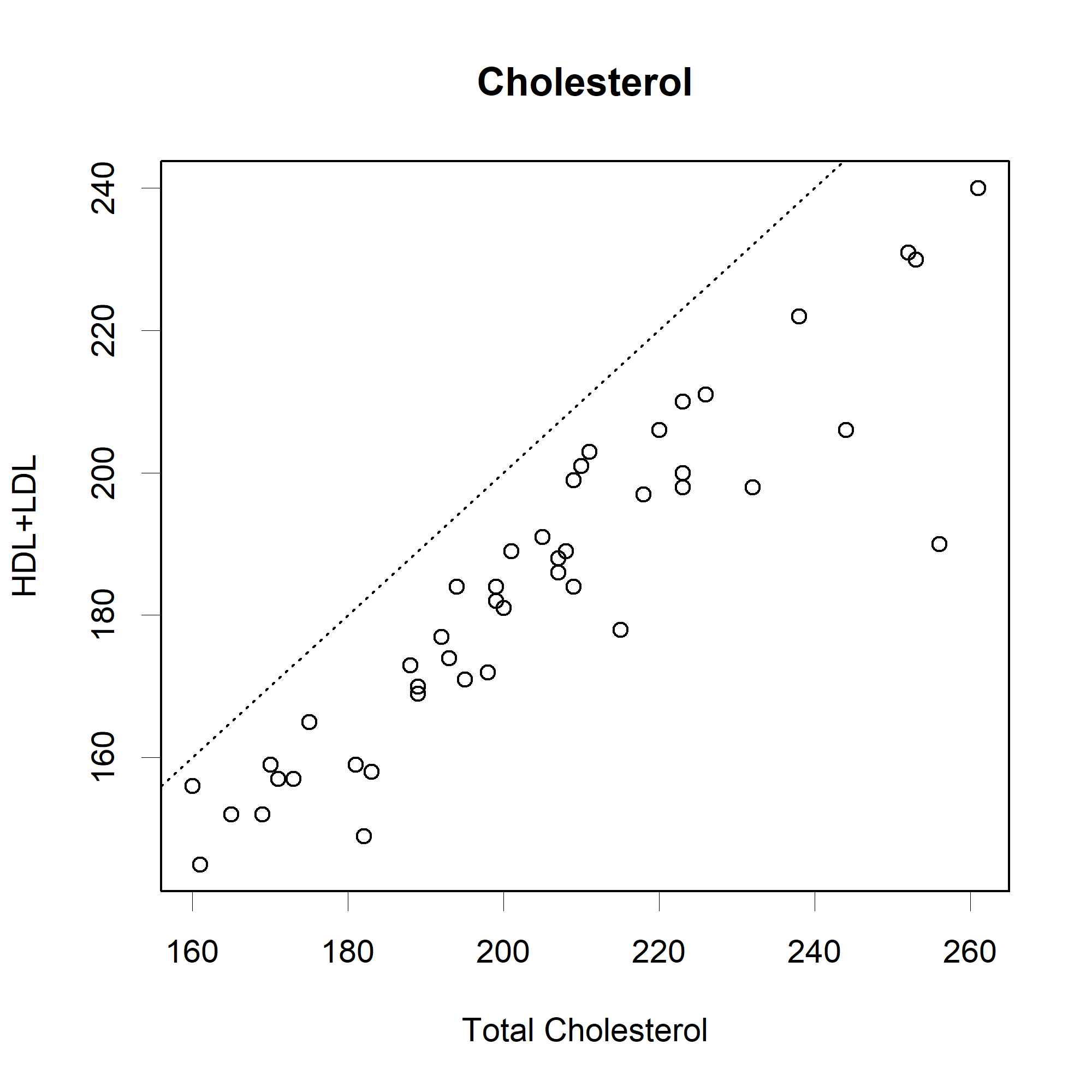

Supplement: S1 File — (ZIP) [file pone.0275066.s001.zip › supporting/fig/cholesterol_measured.png]

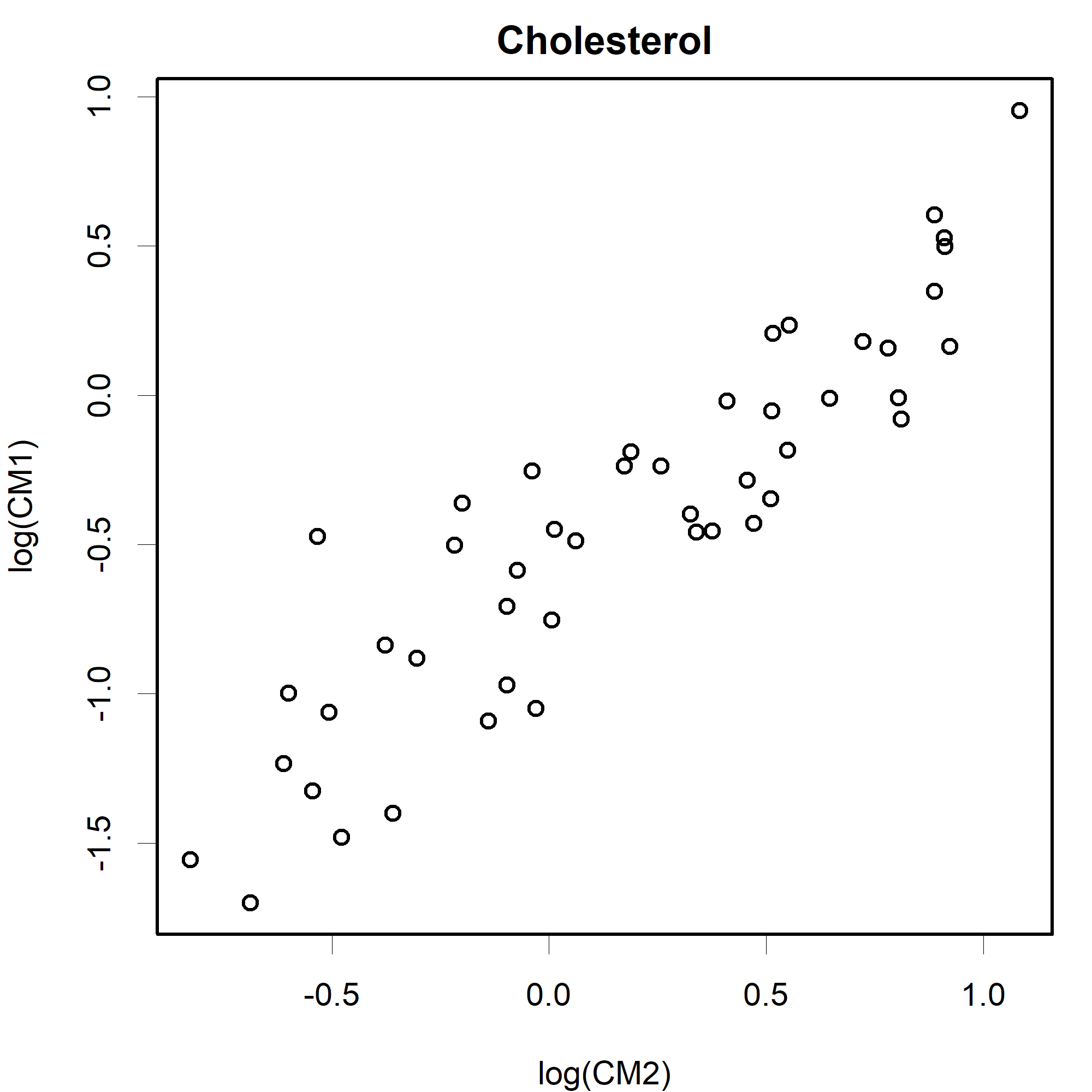

Supplement: S1 File — (ZIP) [file pone.0275066.s001.zip › supporting/fig/CM2CM1.png]

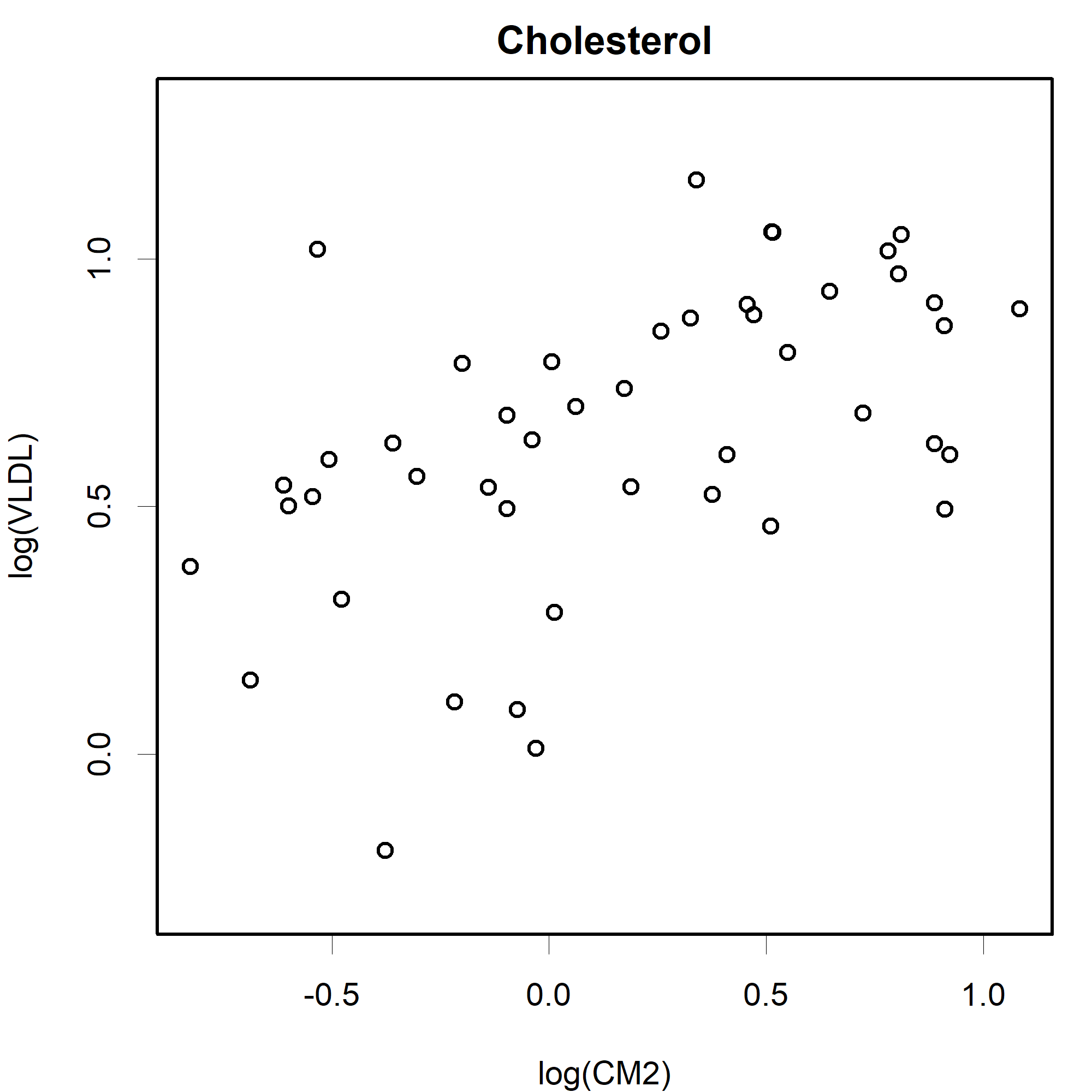

Supplement: S1 File — (ZIP) [file pone.0275066.s001.zip › supporting/fig/CM2VLDL.png]

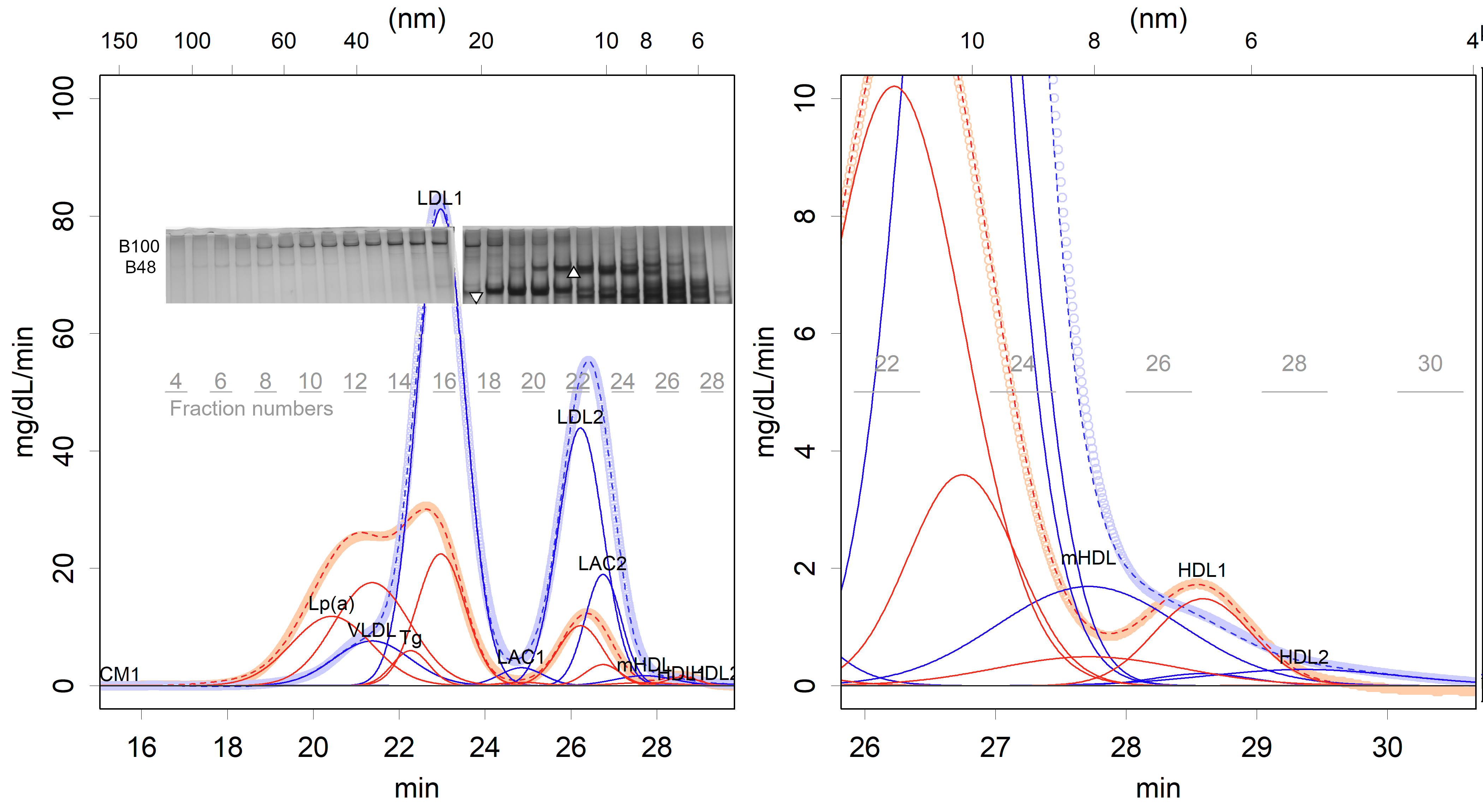

Supplement: S1 File — (ZIP) [file pone.0275066.s001.zip › supporting/fig/Fig1_401.png]

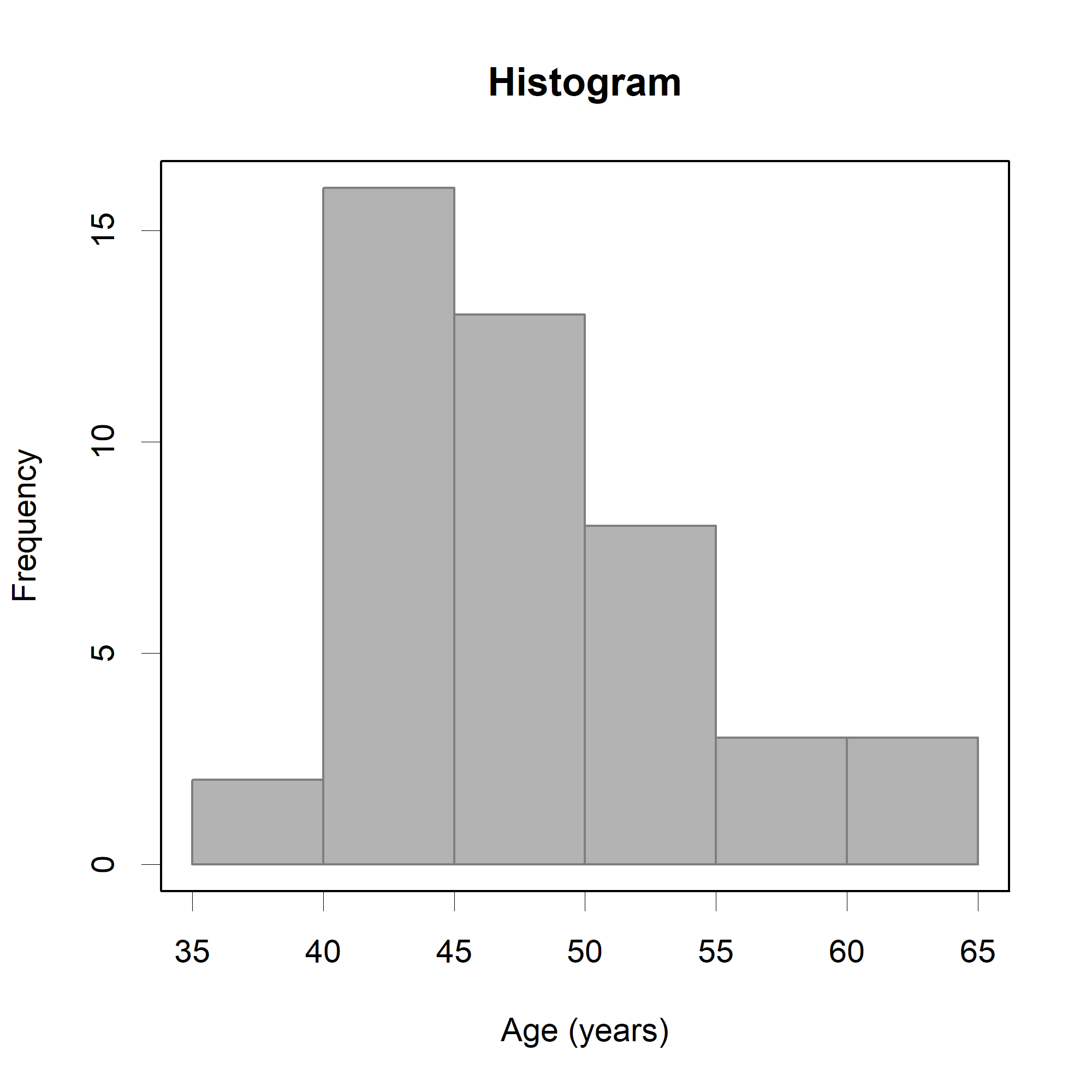

Supplement: S1 File — (ZIP) [file pone.0275066.s001.zip › supporting/fig/histogram_age.png]

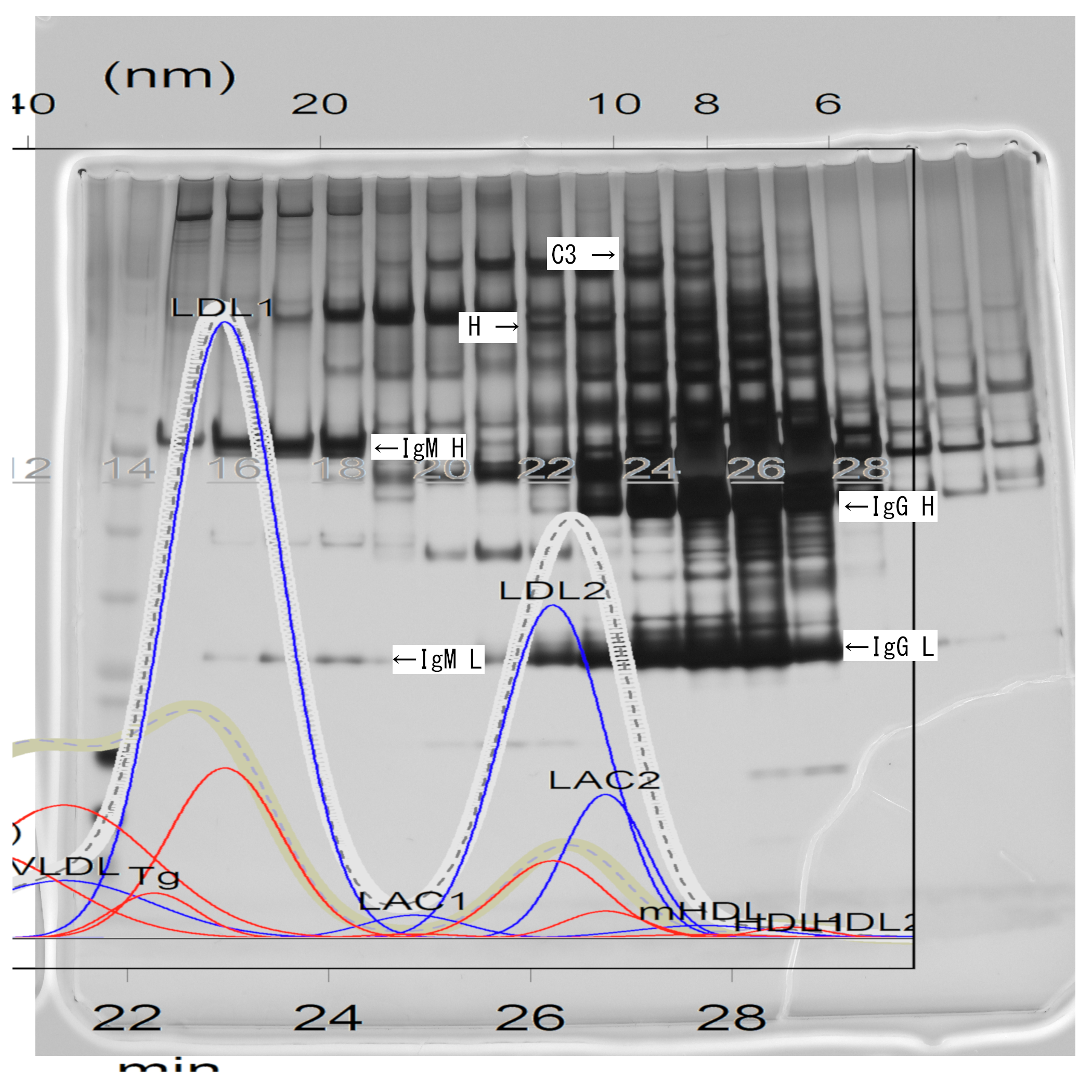

Supplement: S1 File — (ZIP) [file pone.0275066.s001.zip › supporting/fig/kasanee.png]

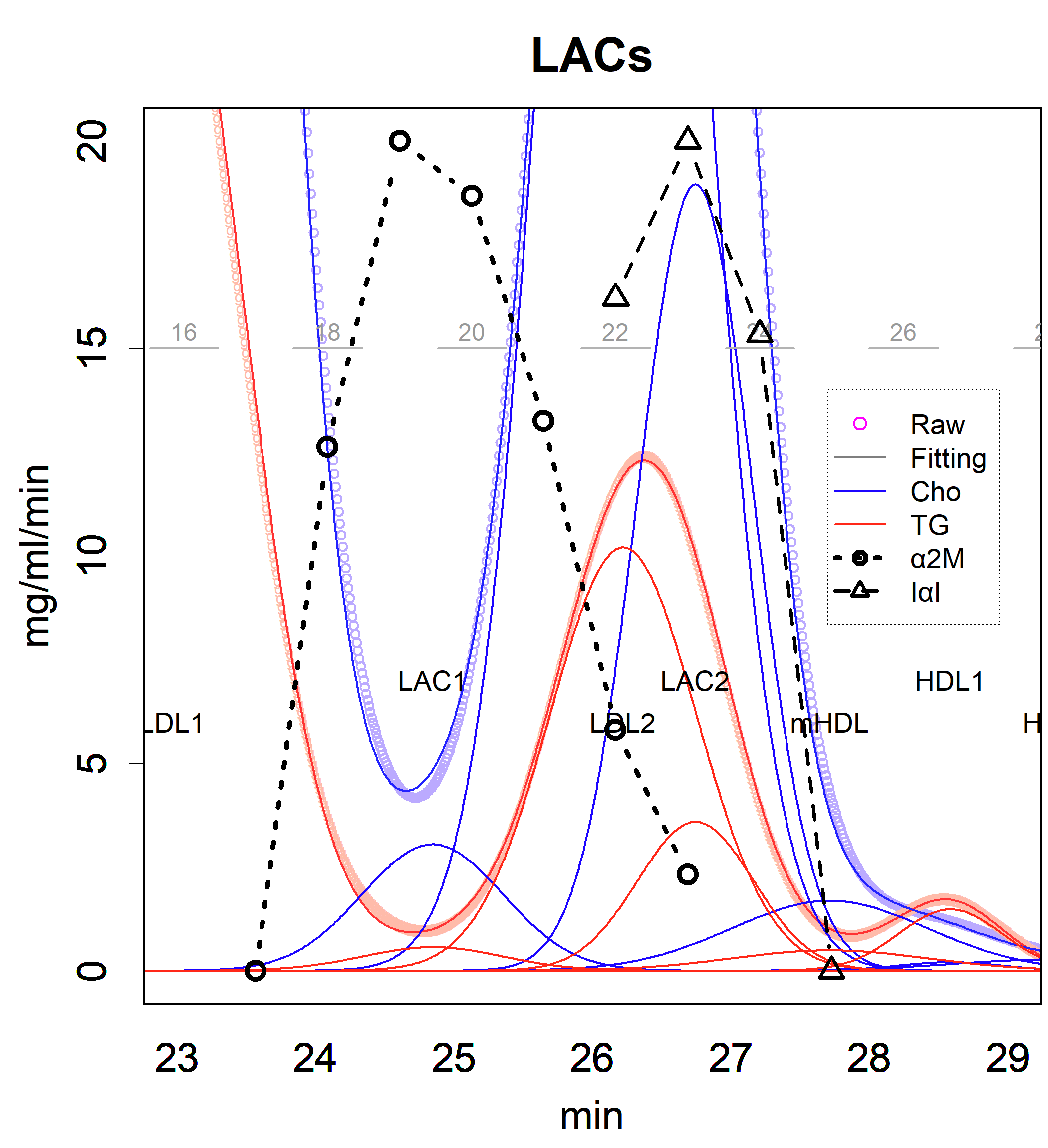

Supplement: S1 File — (ZIP) [file pone.0275066.s001.zip › supporting/fig/LAC_401.png]

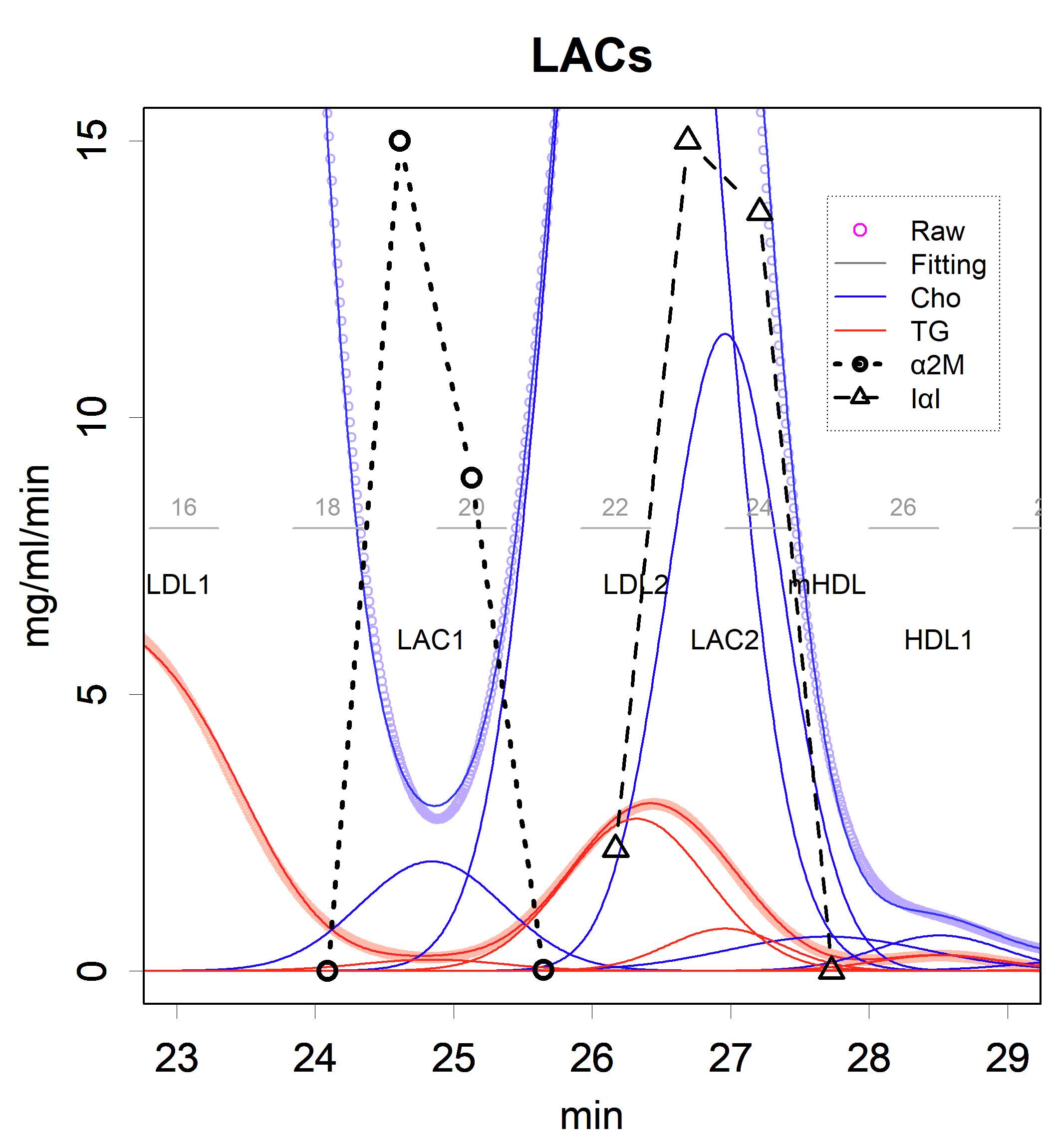

Supplement: S1 File — (ZIP) [file pone.0275066.s001.zip › supporting/fig/LAC_402.png]

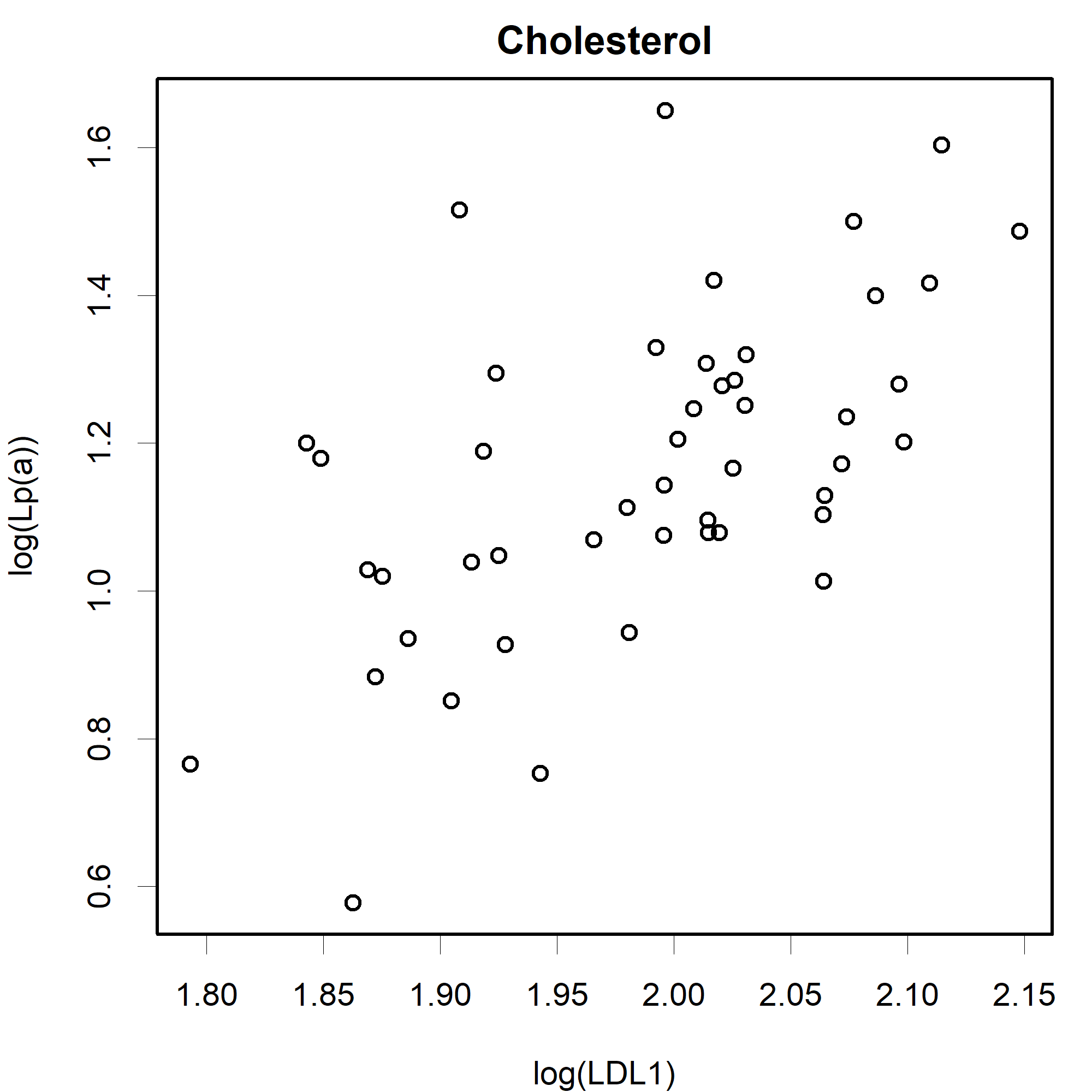

Supplement: S1 File — (ZIP) [file pone.0275066.s001.zip › supporting/fig/LDL1Lpa.png]

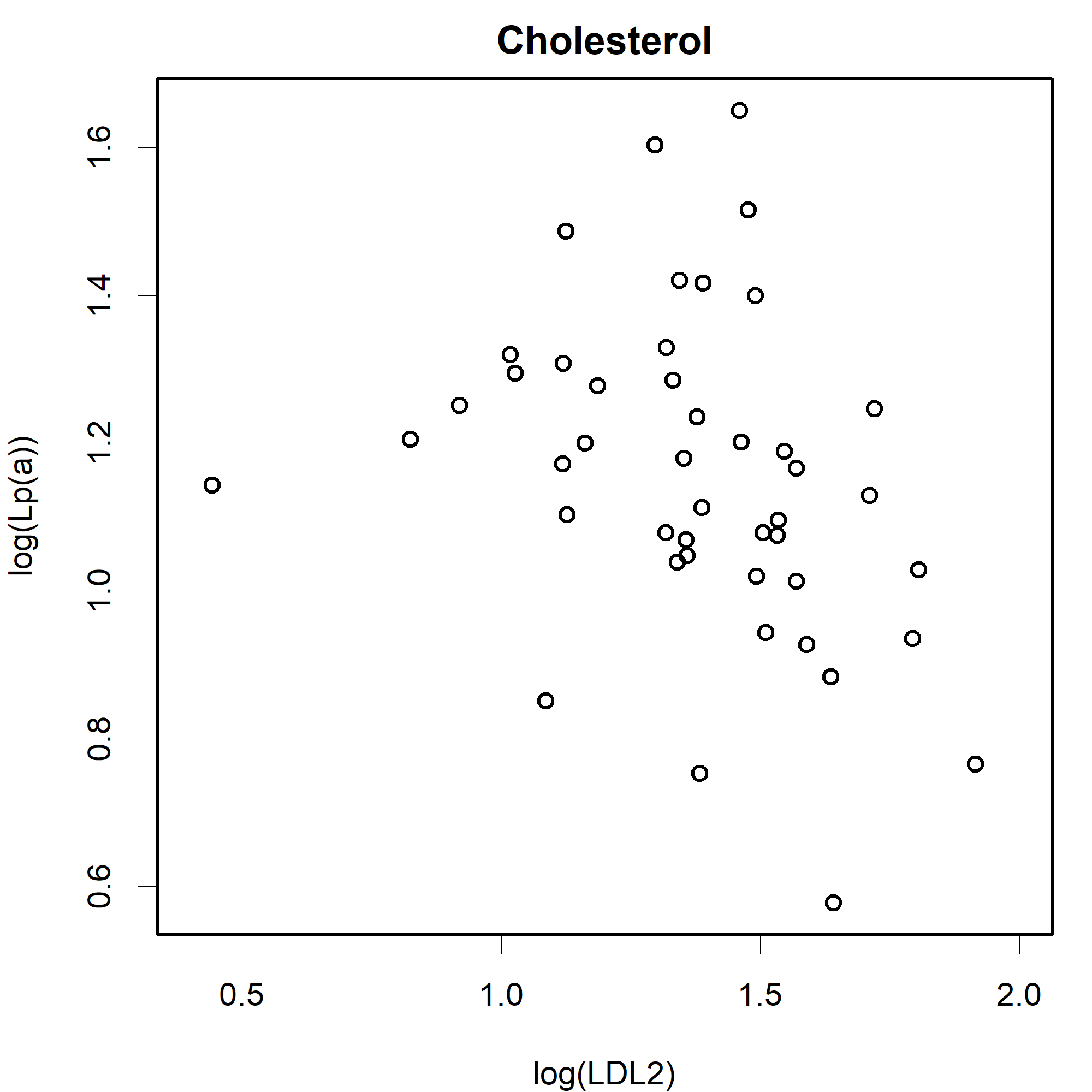

Supplement: S1 File — (ZIP) [file pone.0275066.s001.zip › supporting/fig/LDL2Lpa.png]

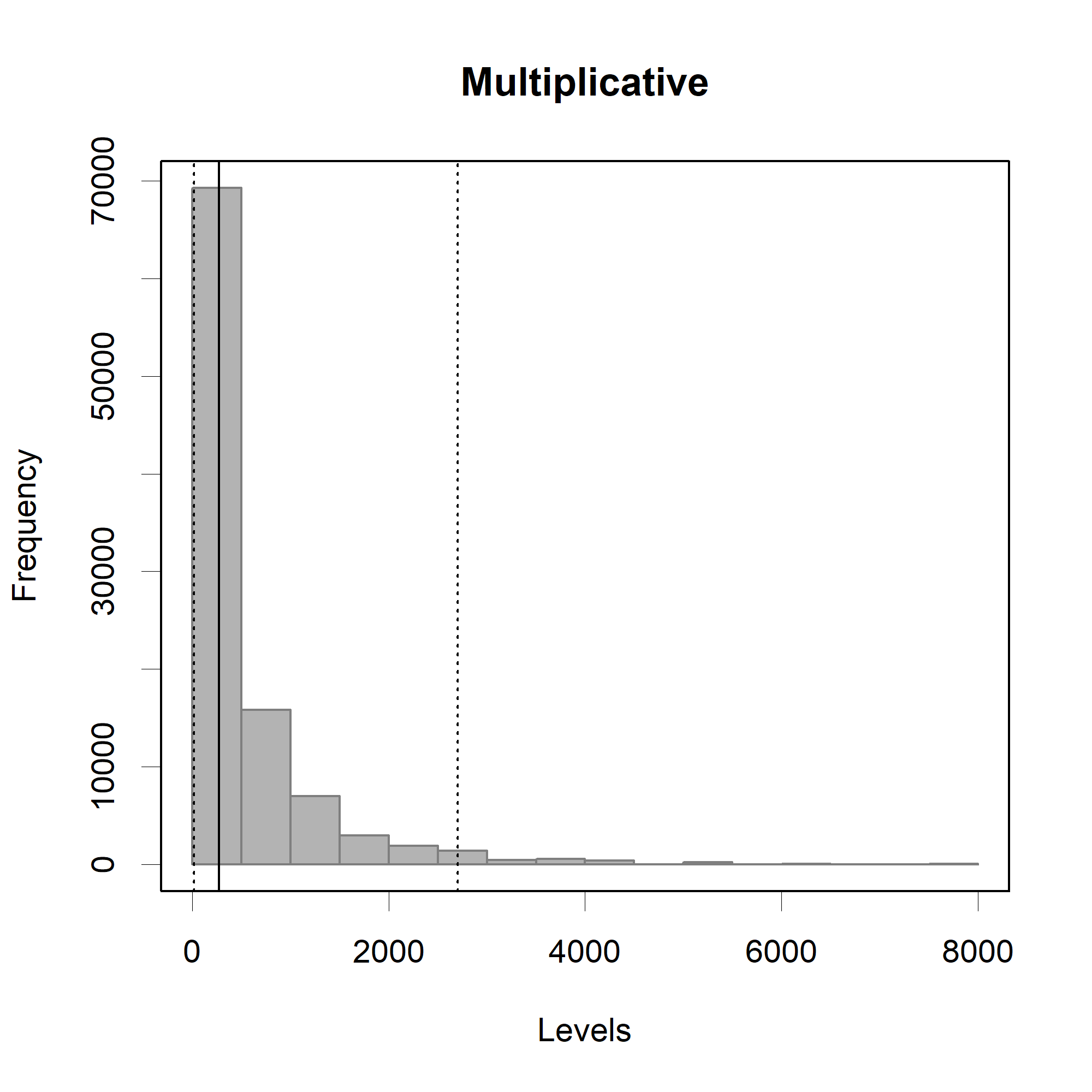

Supplement: S1 File — (ZIP) [file pone.0275066.s001.zip › supporting/fig/log.png]

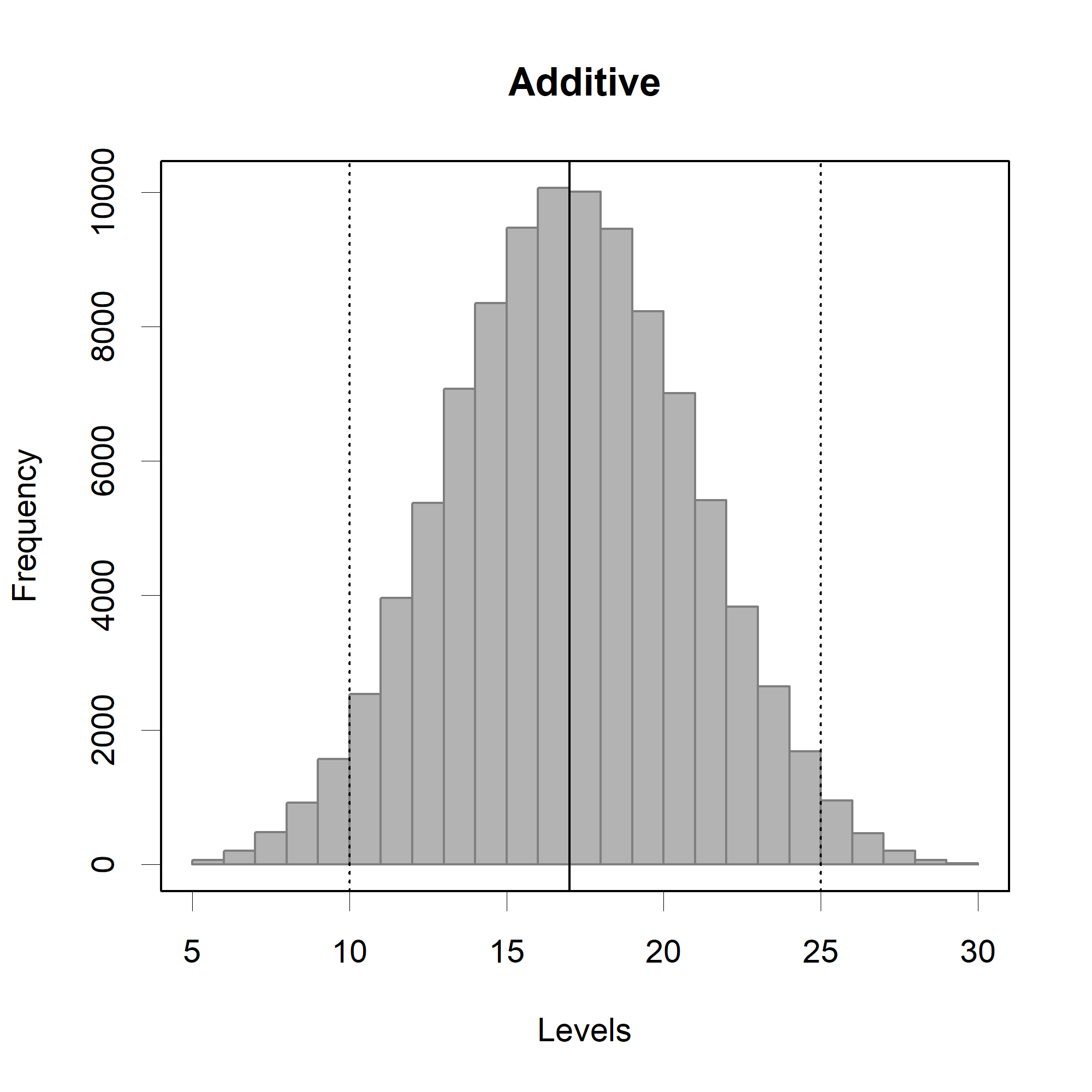

Supplement: S1 File — (ZIP) [file pone.0275066.s001.zip › supporting/fig/nom.png]

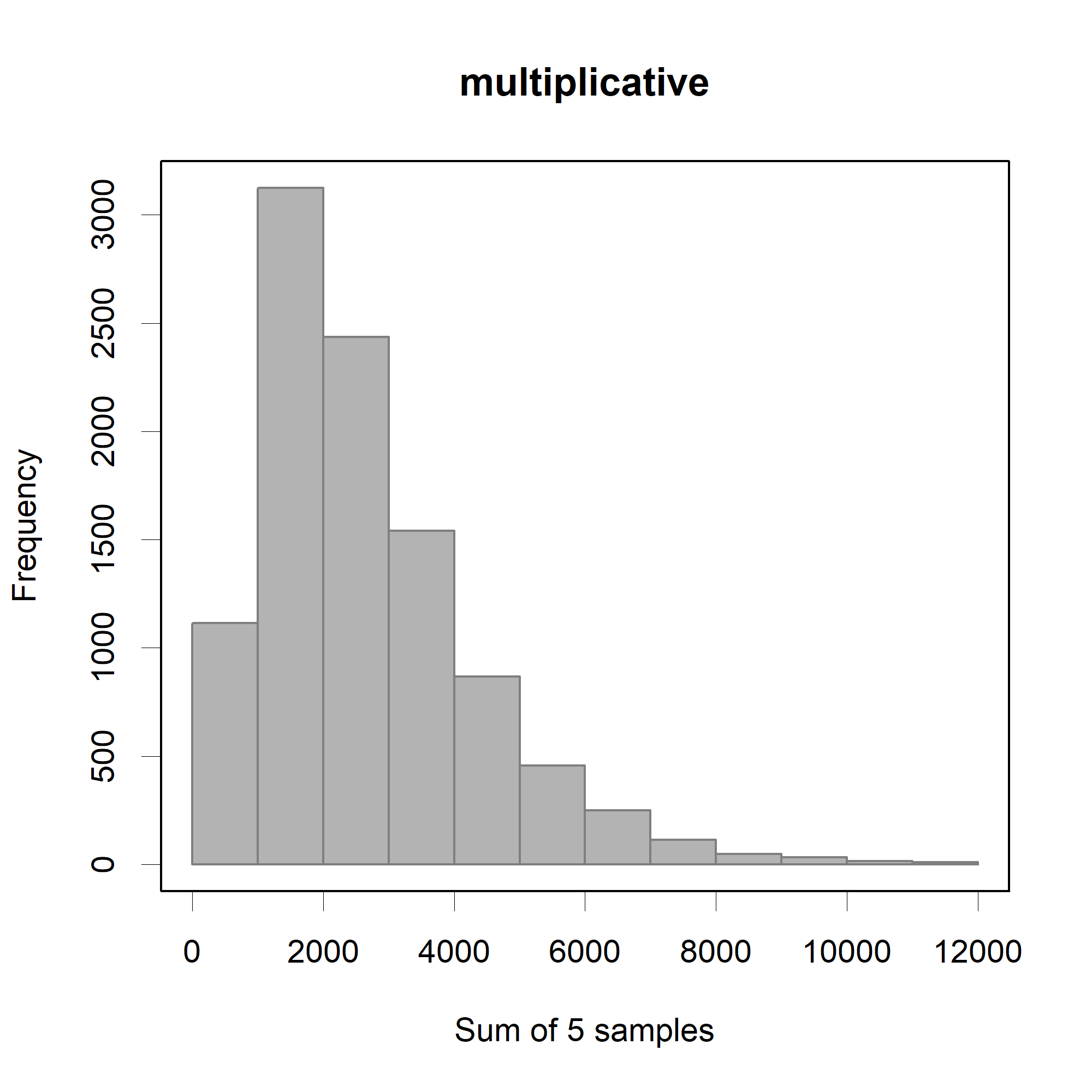

Supplement: S1 File — (ZIP) [file pone.0275066.s001.zip › supporting/fig/sample.png]

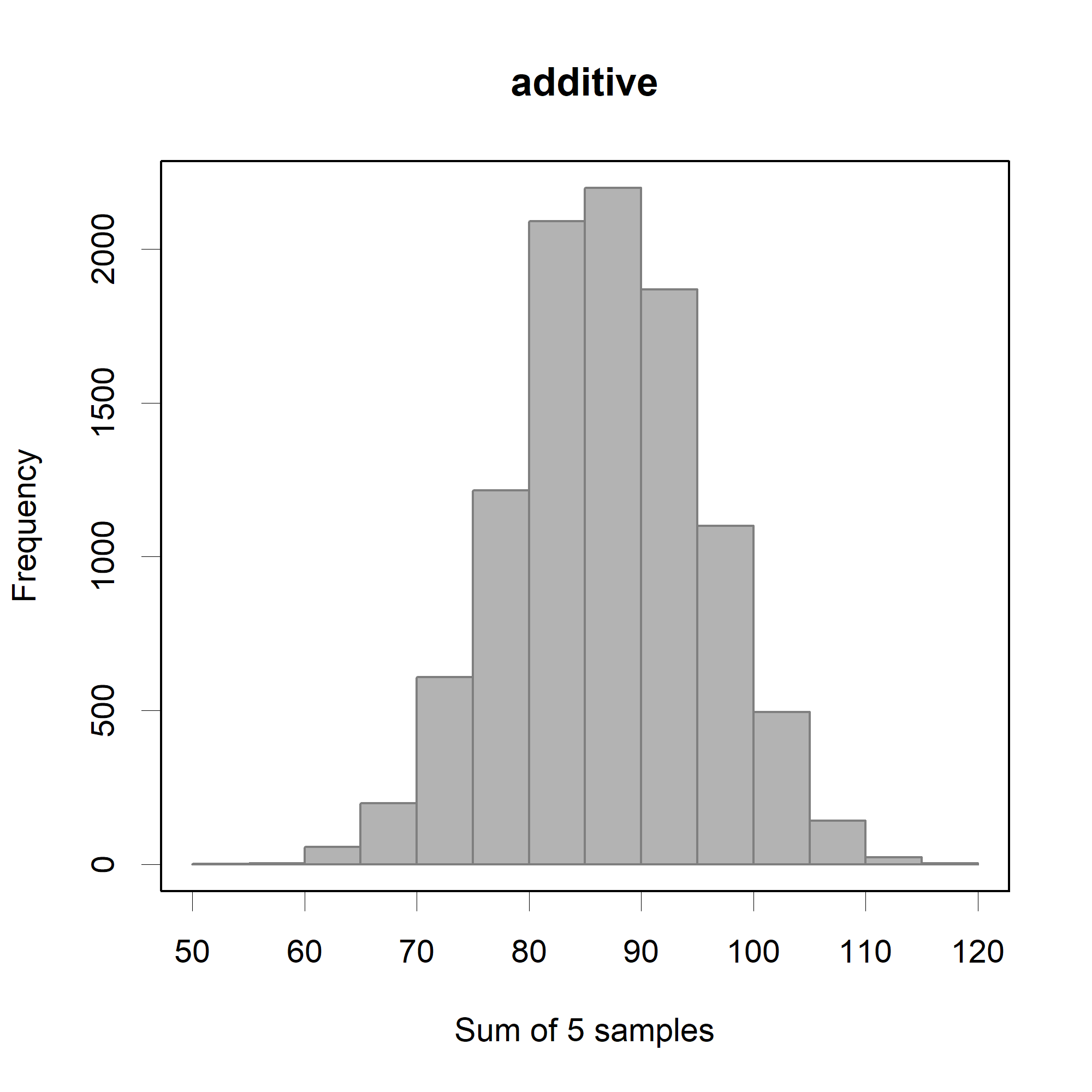

Supplement: S1 File — (ZIP) [file pone.0275066.s001.zip › supporting/fig/samplea.png]

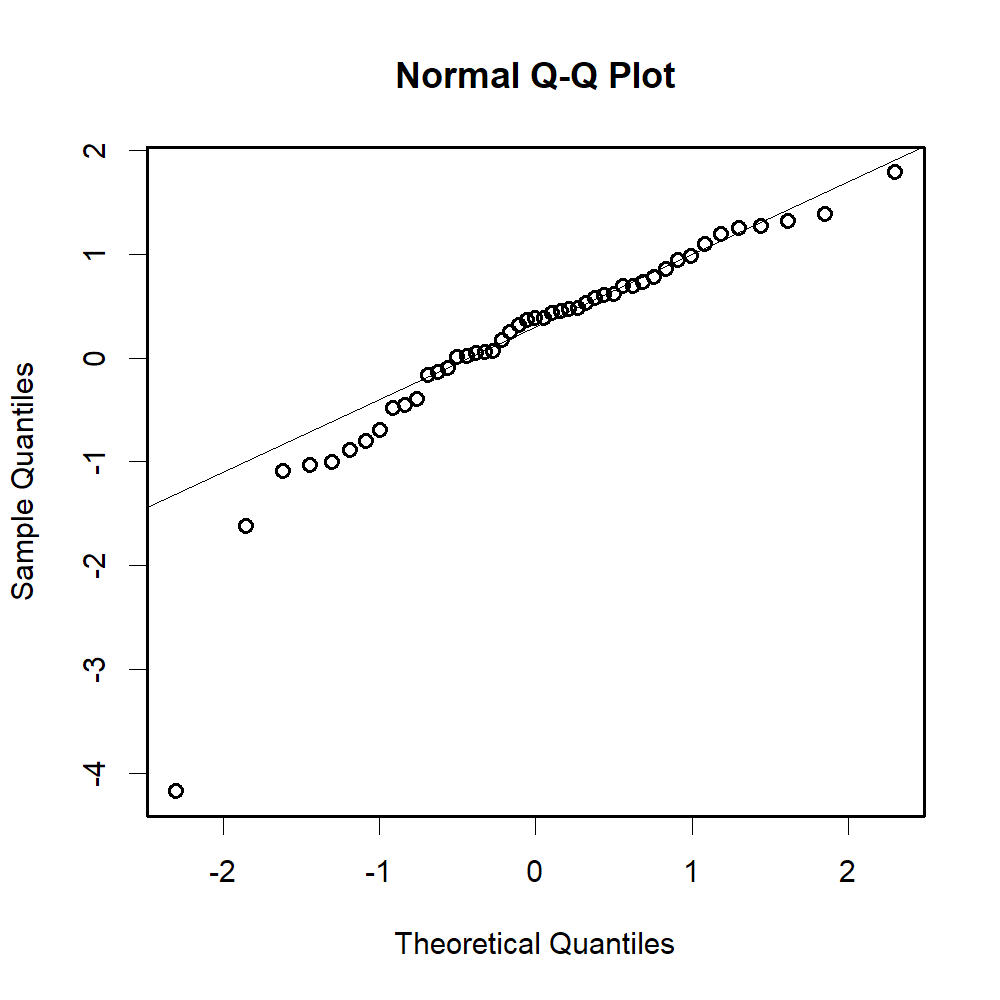

Supplement: S1 File — (ZIP) [file pone.0275066.s001.zip › supporting/fig/TG/CM1.png]

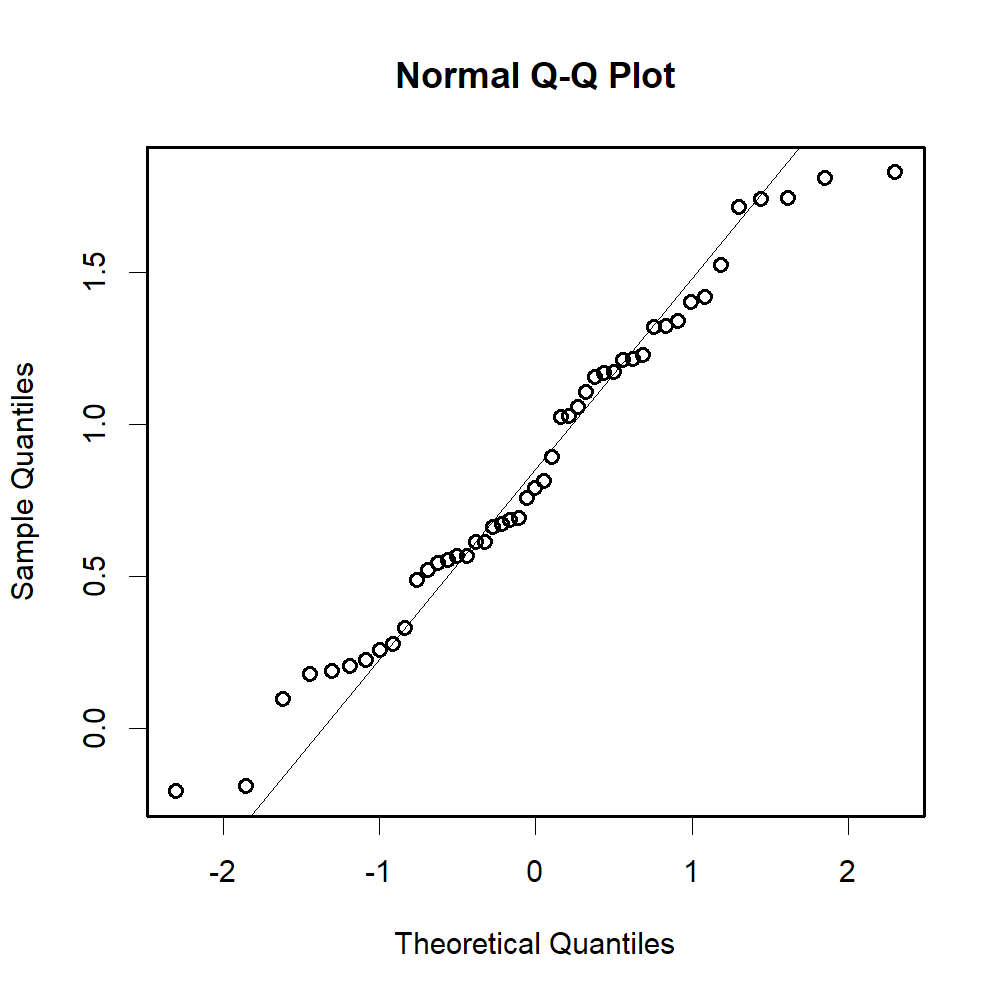

Supplement: S1 File — (ZIP) [file pone.0275066.s001.zip › supporting/fig/TG/CM2.png]

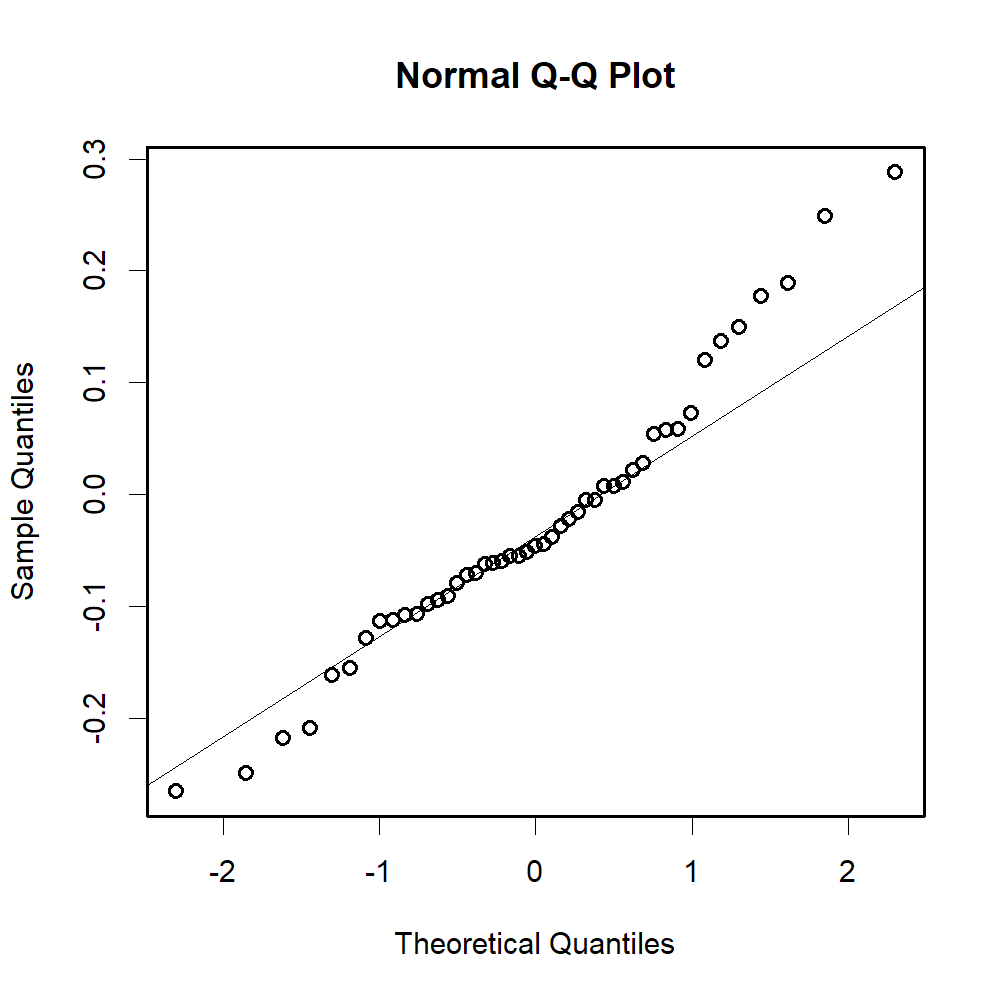

Supplement: S1 File — (ZIP) [file pone.0275066.s001.zip › supporting/fig/TG/HDL1.png]

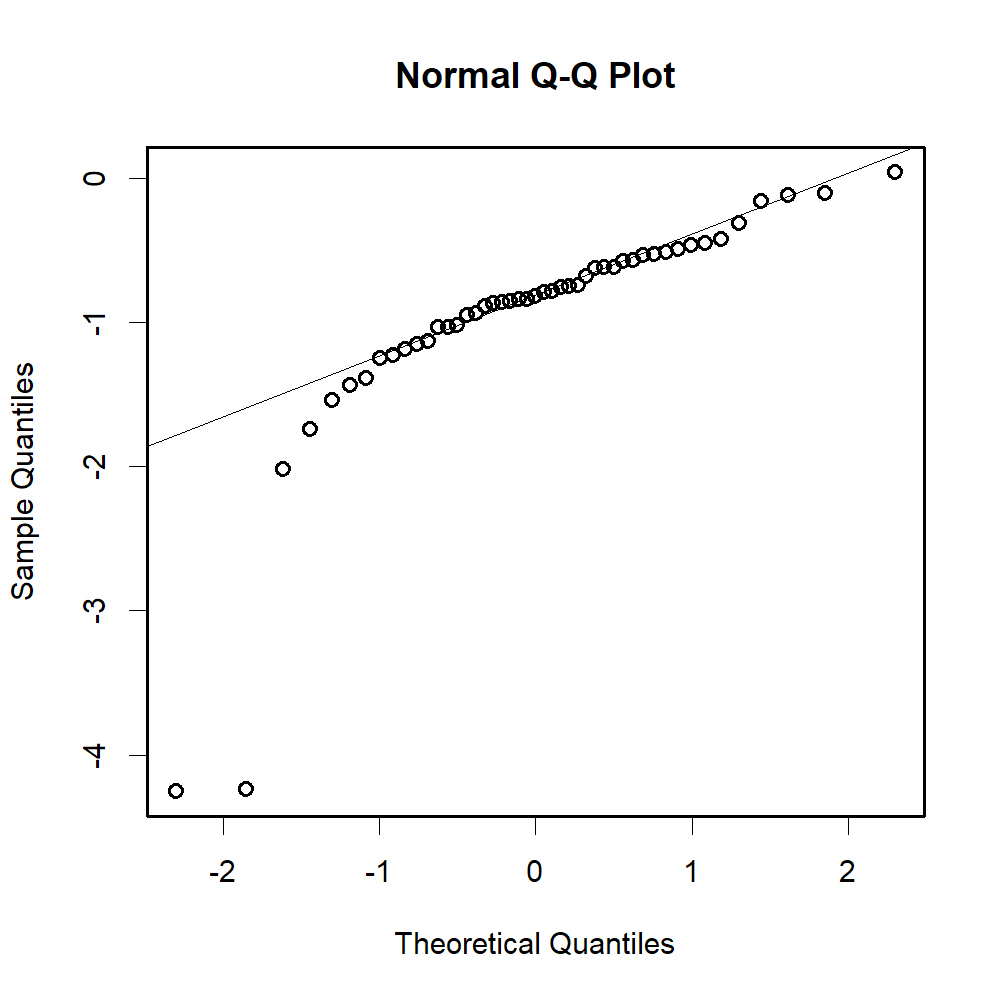

Supplement: S1 File — (ZIP) [file pone.0275066.s001.zip › supporting/fig/TG/HDL2.png]

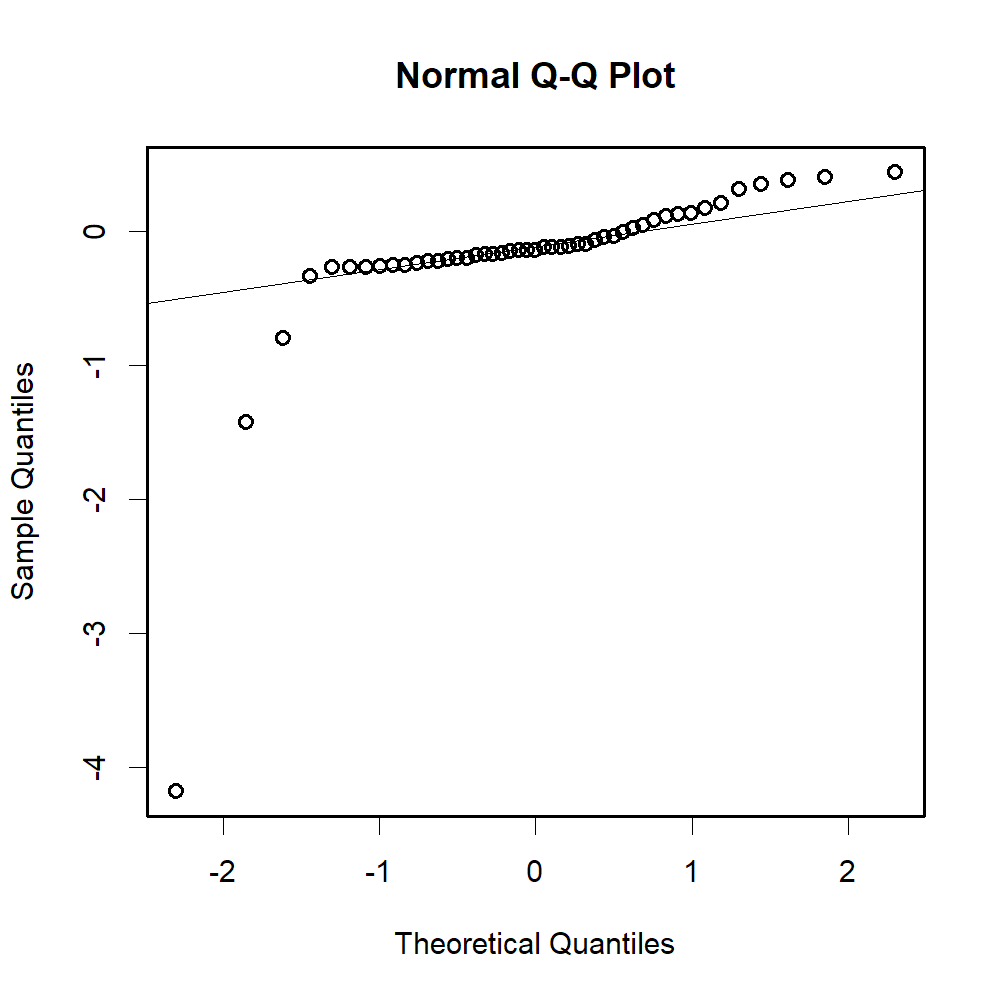

Supplement: S1 File — (ZIP) [file pone.0275066.s001.zip › supporting/fig/TG/LAC1.png]

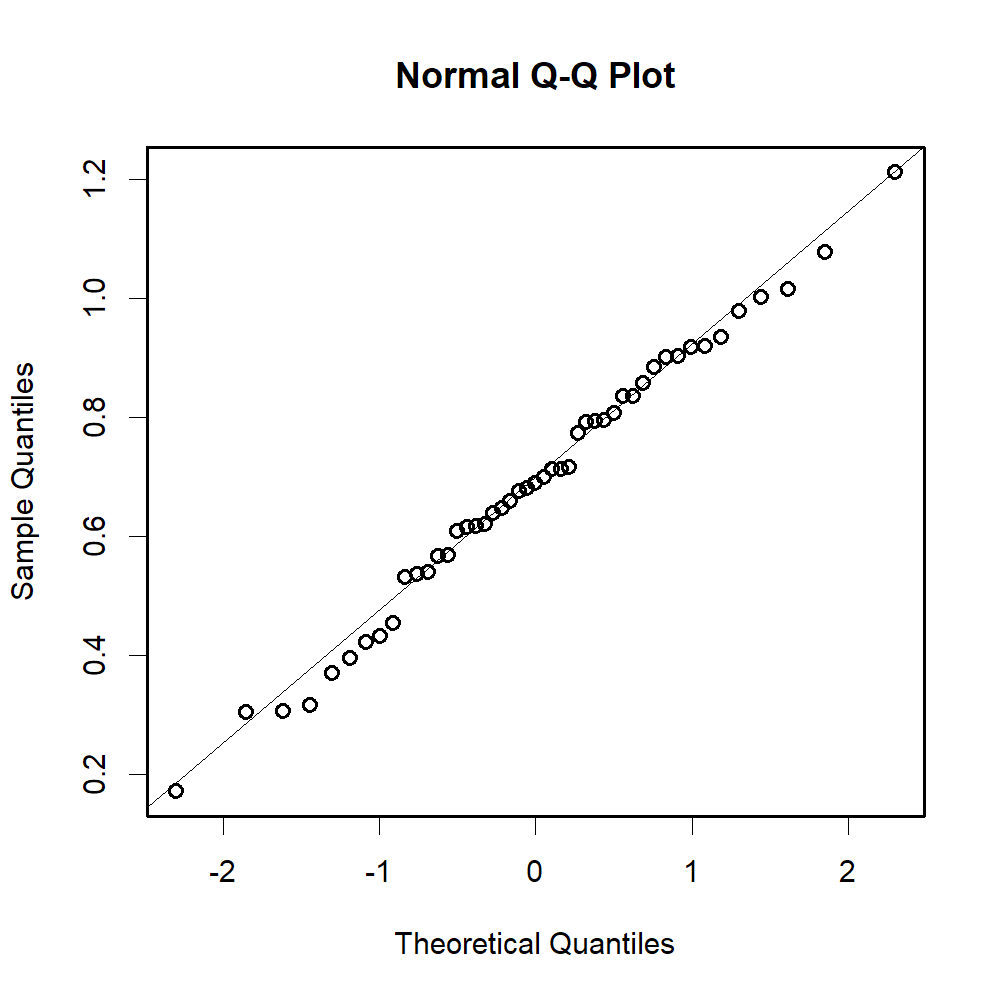

Supplement: S1 File — (ZIP) [file pone.0275066.s001.zip › supporting/fig/TG/LAC2.png]

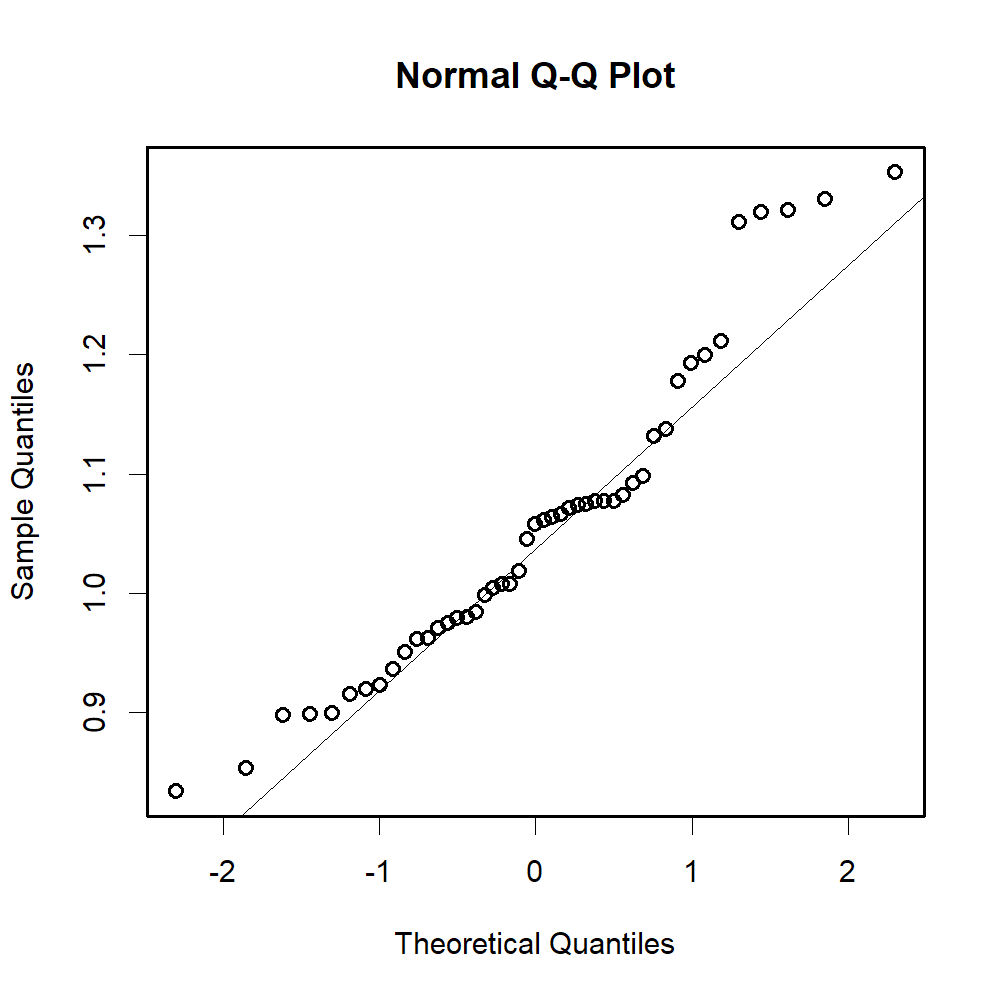

Supplement: S1 File — (ZIP) [file pone.0275066.s001.zip › supporting/fig/TG/LDL1.png]

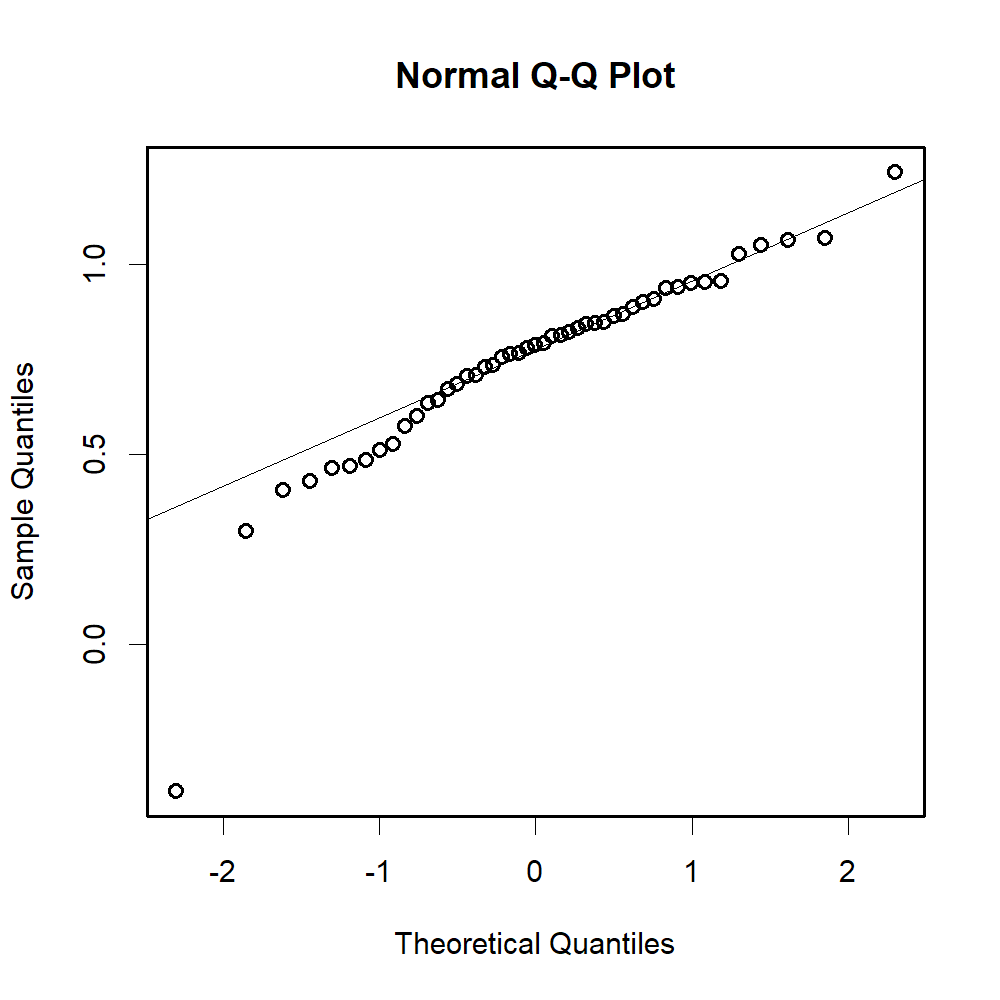

Supplement: S1 File — (ZIP) [file pone.0275066.s001.zip › supporting/fig/TG/LDL2.png]

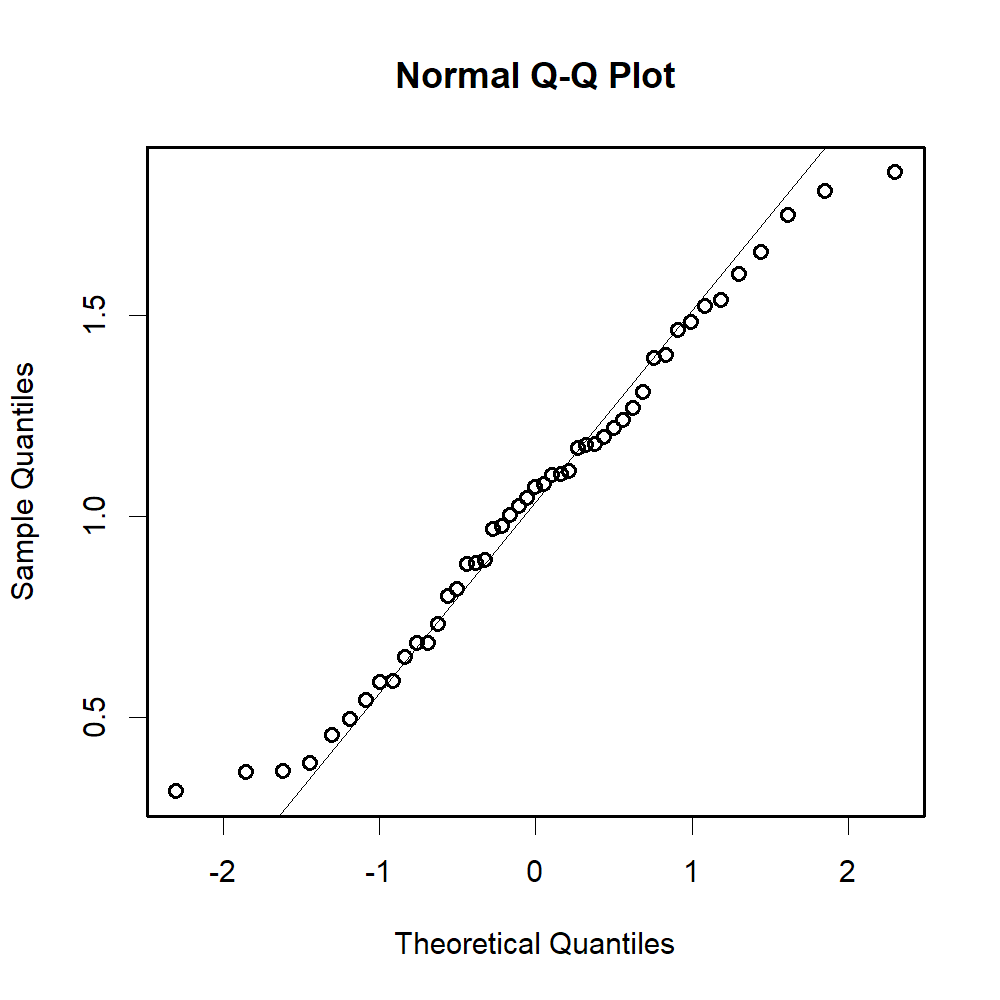

Supplement: S1 File — (ZIP) [file pone.0275066.s001.zip › supporting/fig/TG/Lp(a).png]

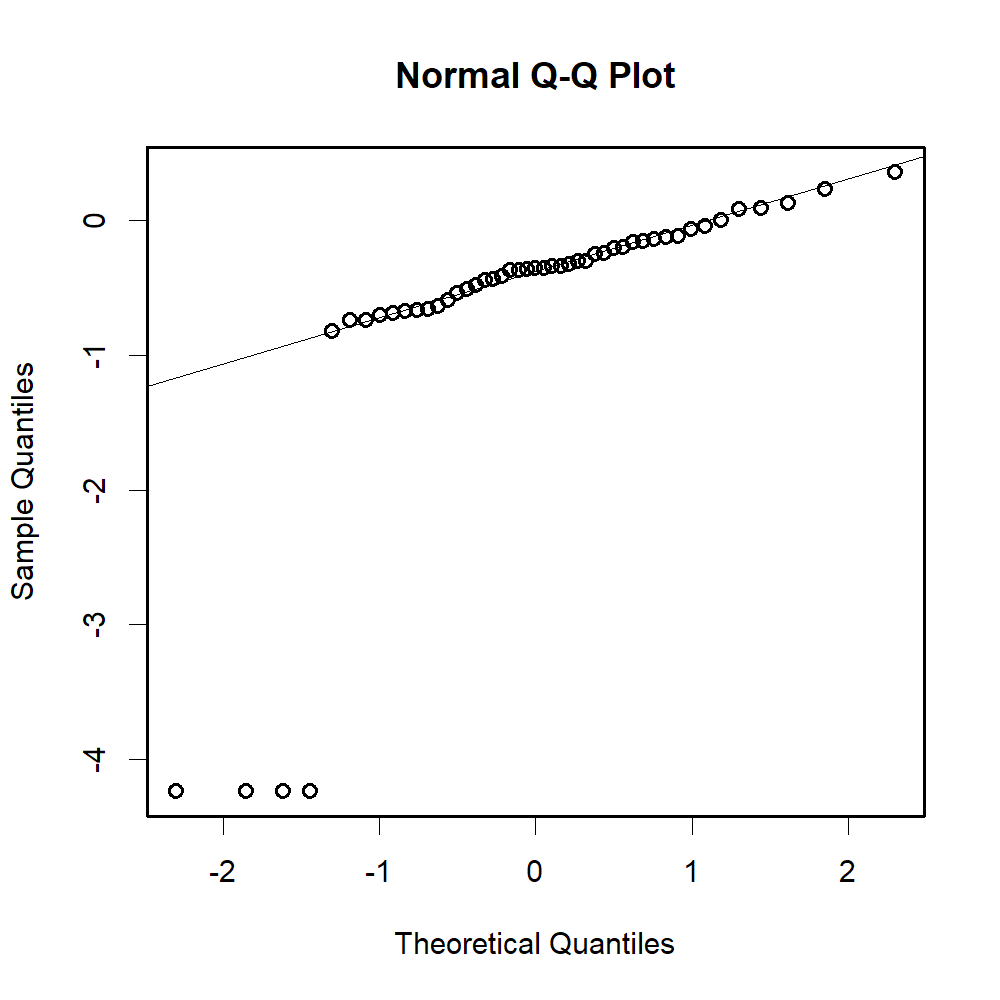

Supplement: S1 File — (ZIP) [file pone.0275066.s001.zip › supporting/fig/TG/mHDL.png]

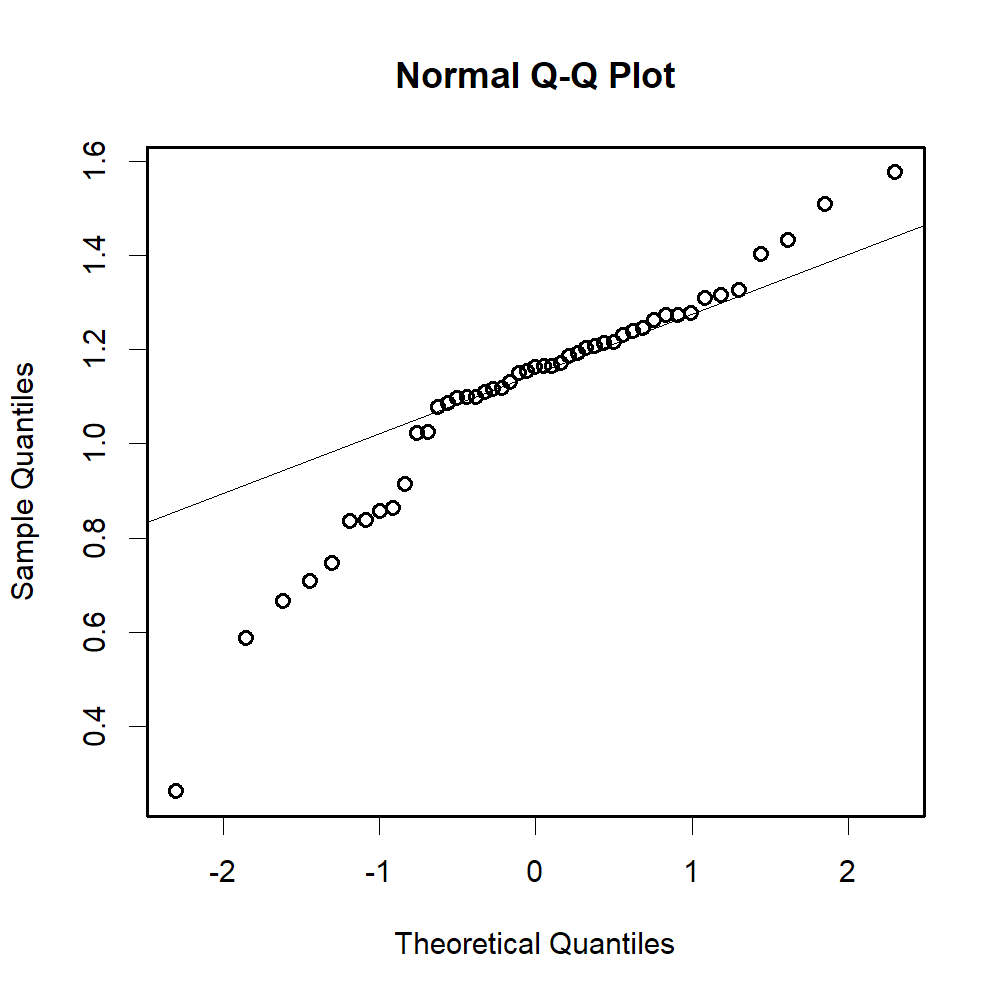

Supplement: S1 File — (ZIP) [file pone.0275066.s001.zip › supporting/fig/TG/Tg.png]

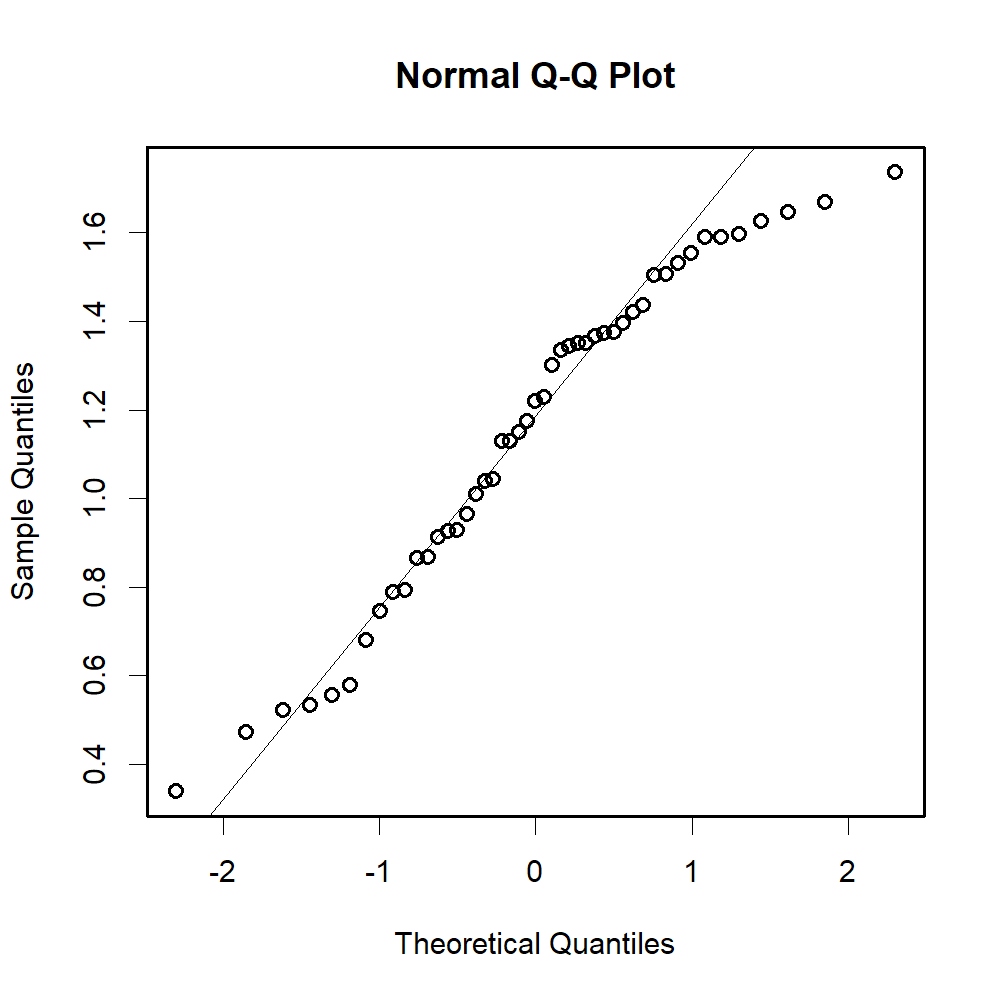

Supplement: S1 File — (ZIP) [file pone.0275066.s001.zip › supporting/fig/TG/VLDL.png]

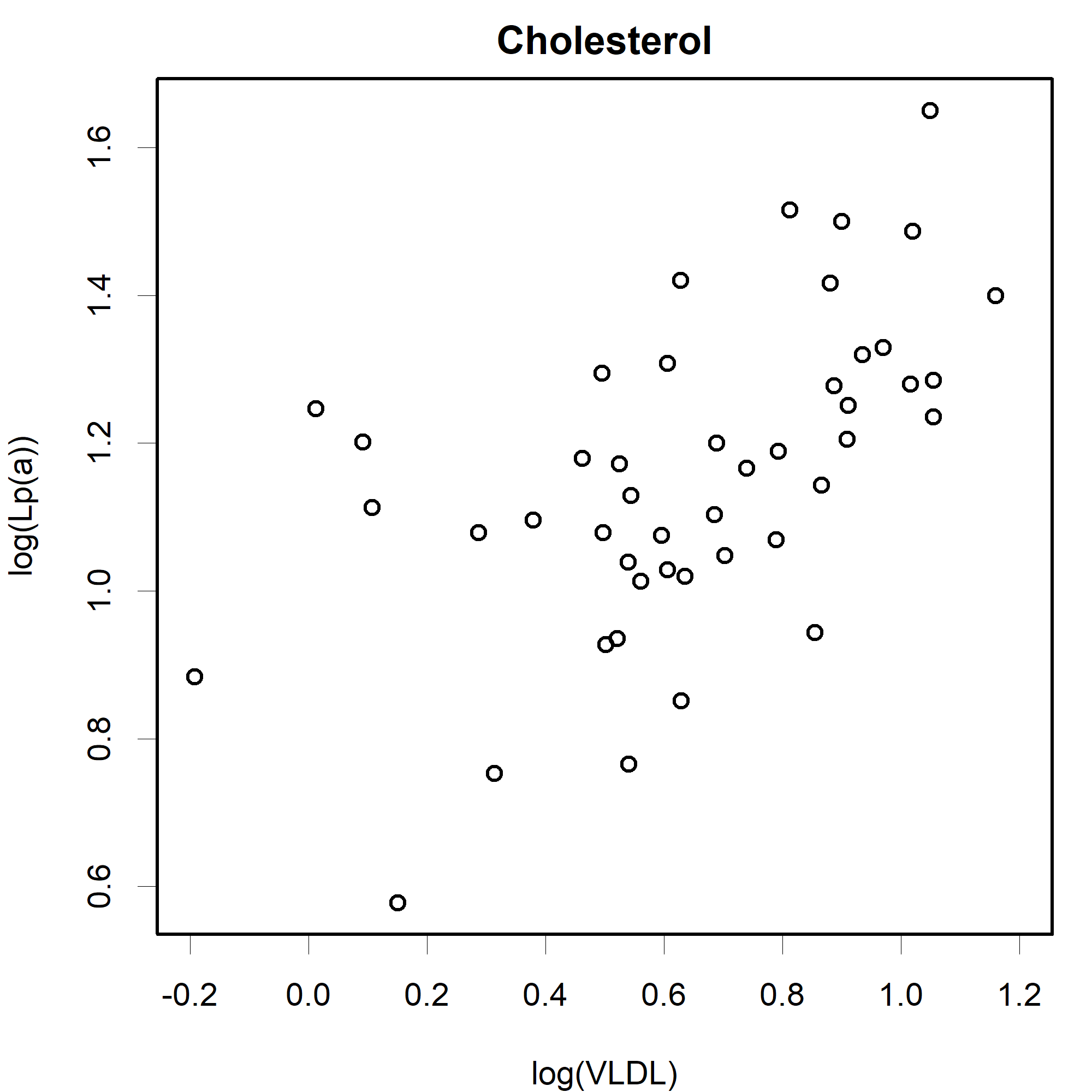

Supplement: S1 File — (ZIP) [file pone.0275066.s001.zip › supporting/fig/VLDLLpa.png]

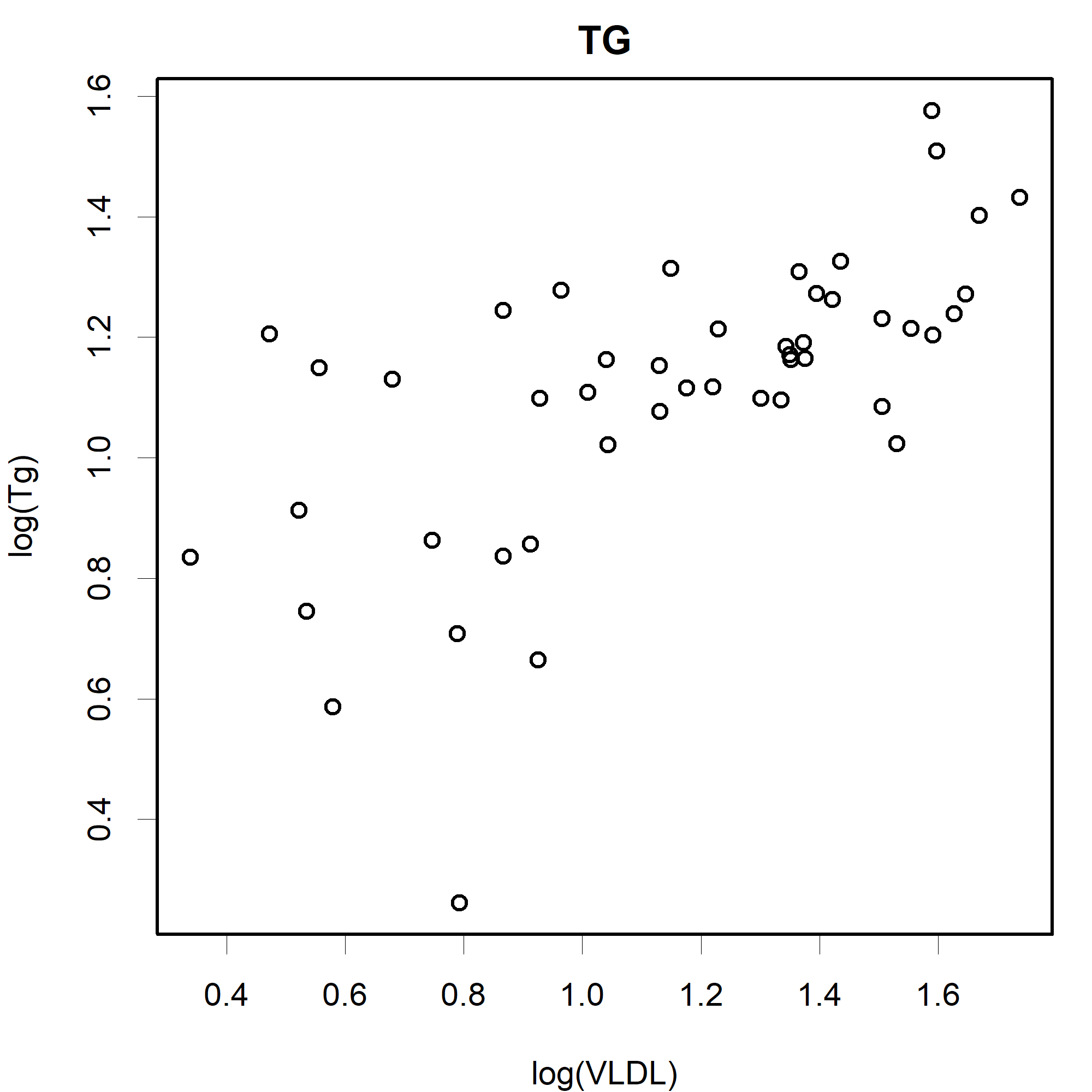

Supplement: S1 File — (ZIP) [file pone.0275066.s001.zip › supporting/fig/VLDLTG.png]

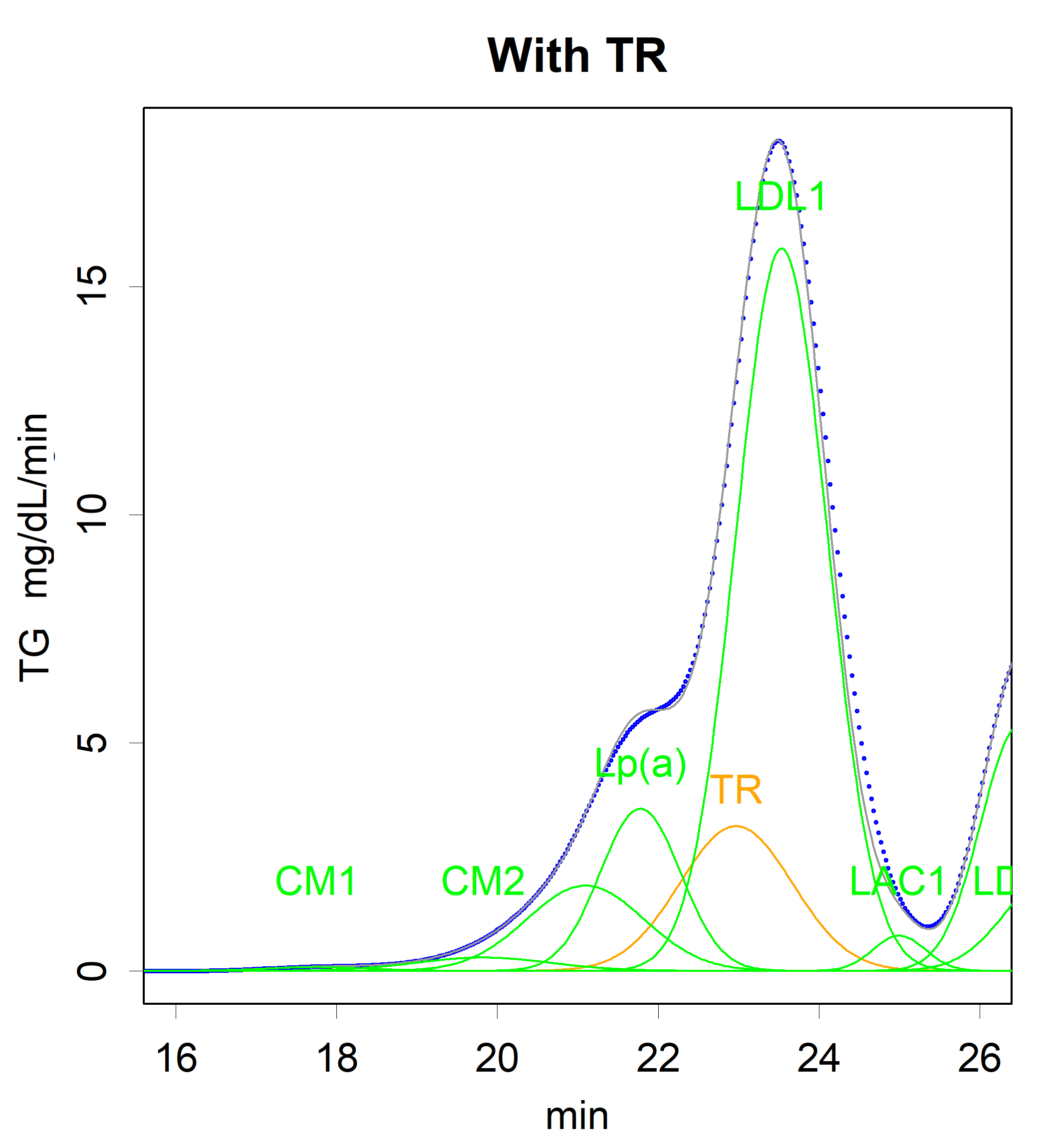

Supplement: S1 File — (ZIP) [file pone.0275066.s001.zip › supporting/fig/withoutTG/with1009.png]

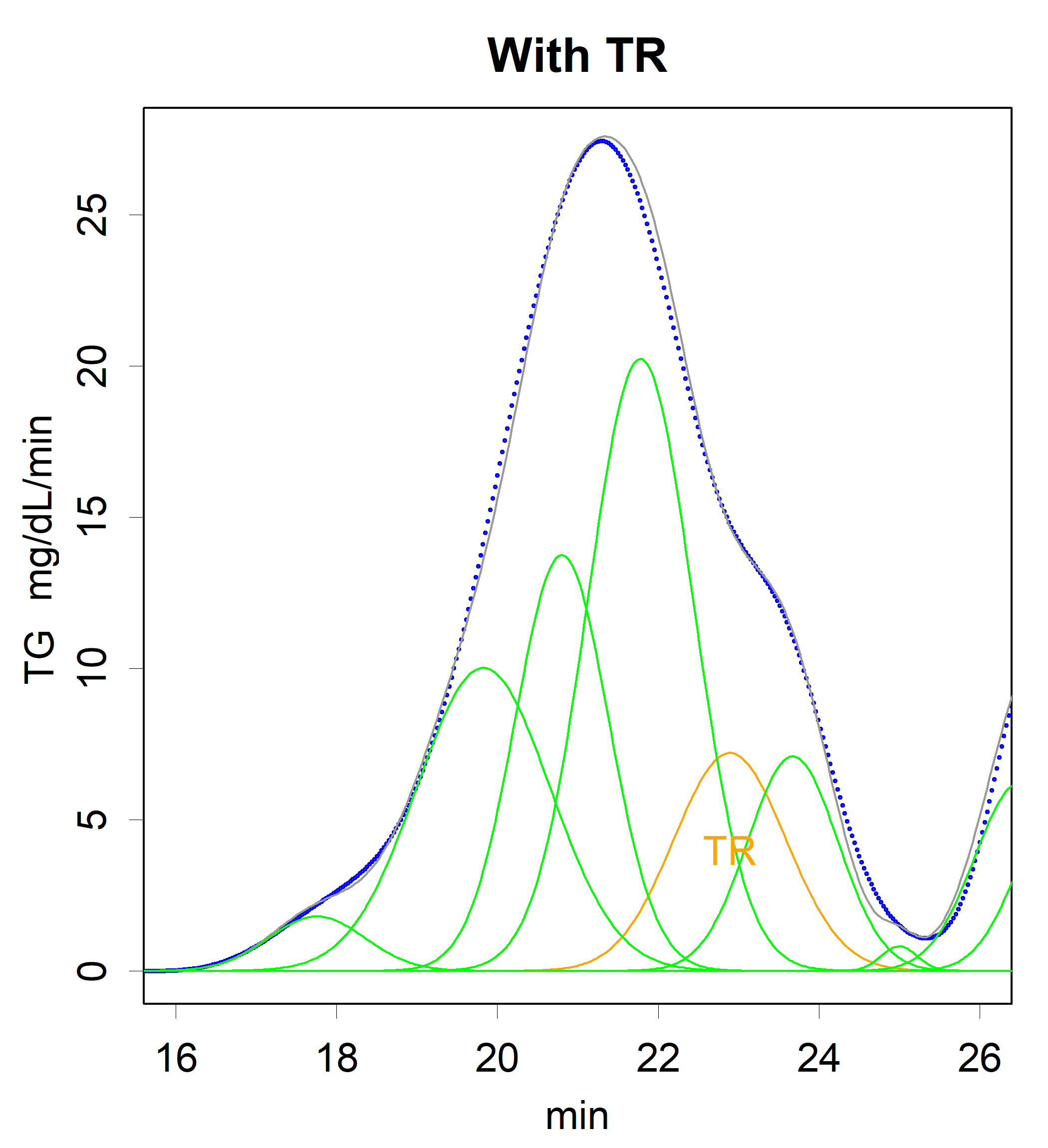

Supplement: S1 File — (ZIP) [file pone.0275066.s001.zip › supporting/fig/withoutTG/with1010.png]

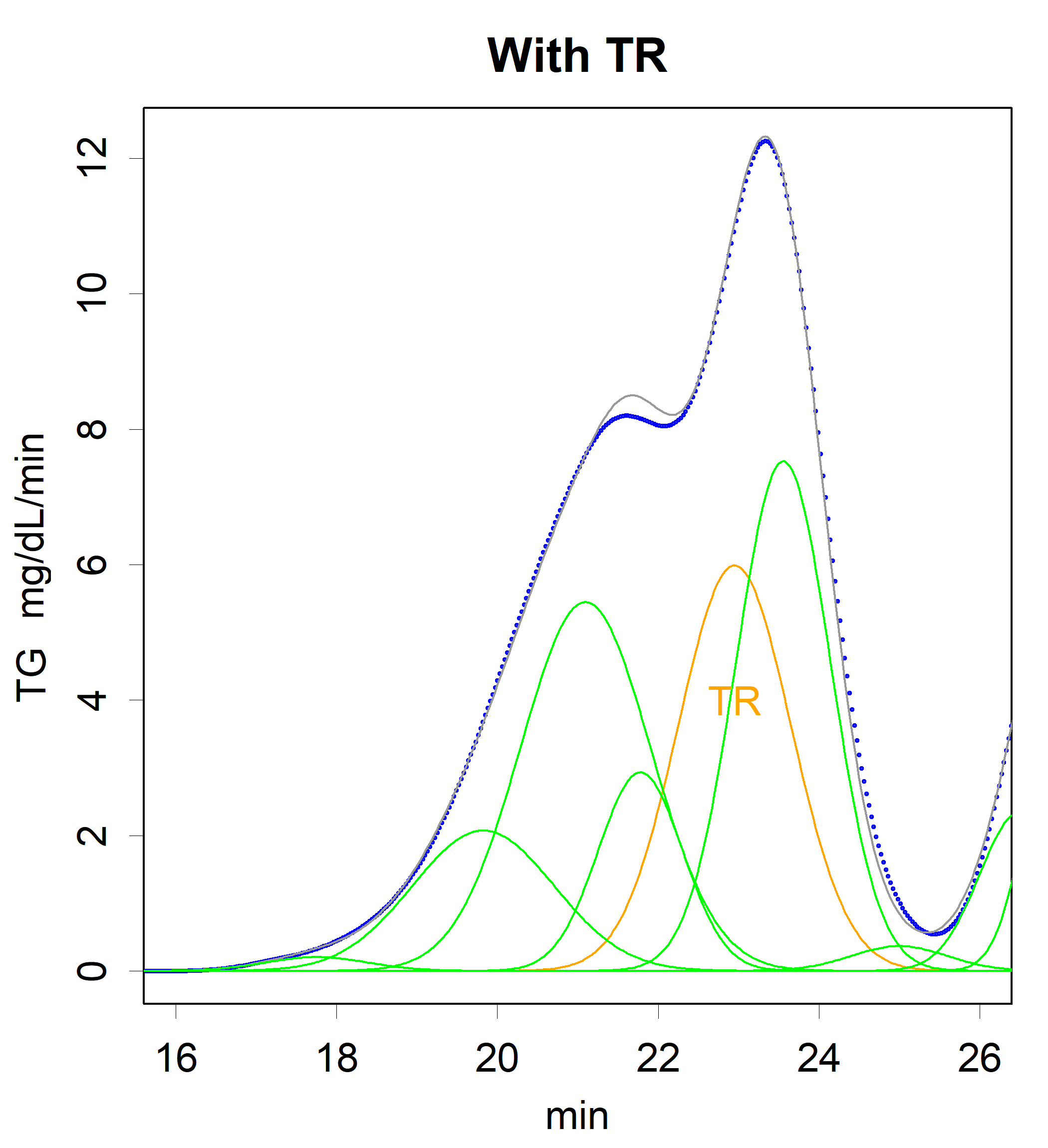

Supplement: S1 File — (ZIP) [file pone.0275066.s001.zip › supporting/fig/withoutTG/with1011.png]

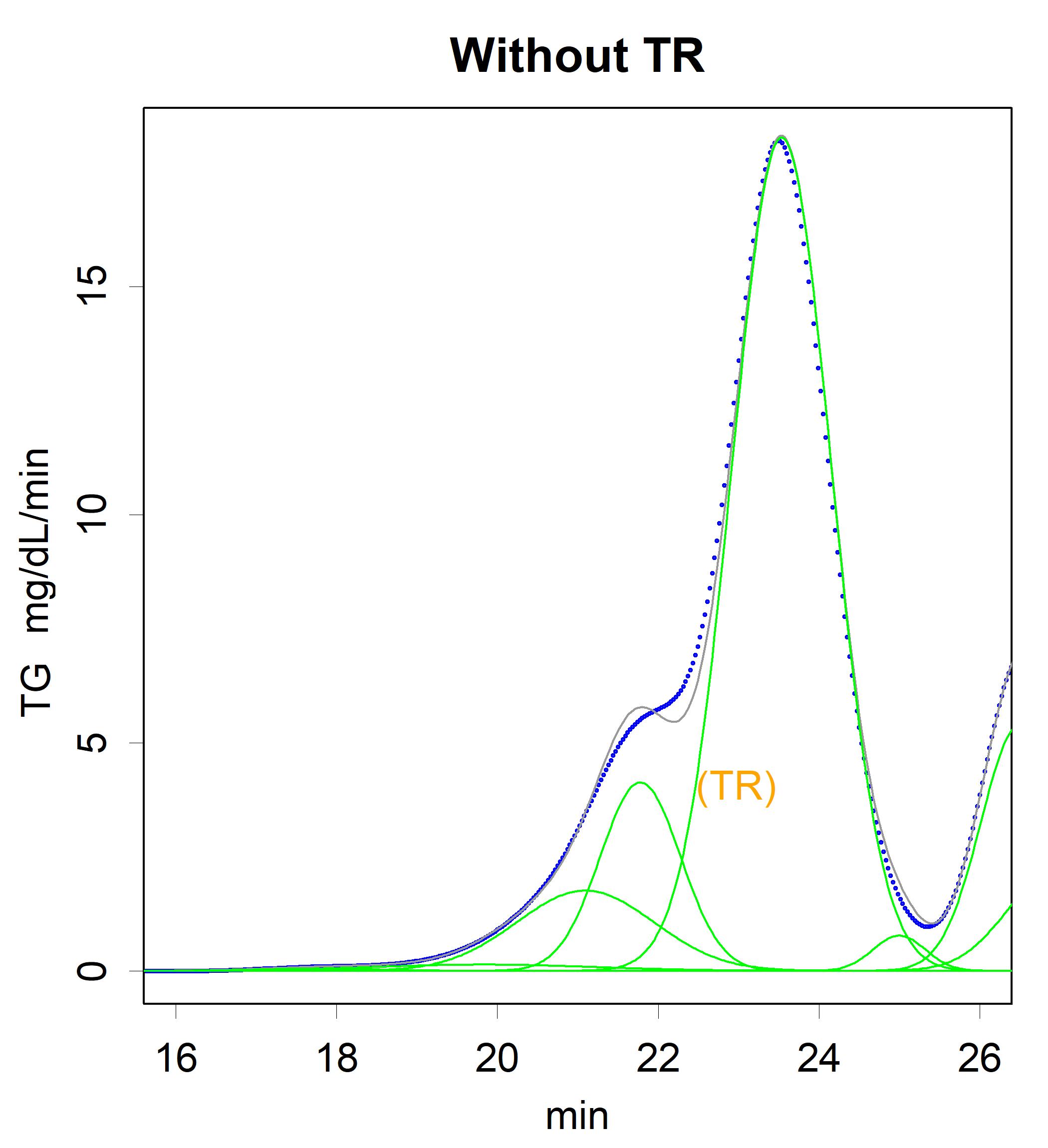

Supplement: S1 File — (ZIP) [file pone.0275066.s001.zip › supporting/fig/withoutTG/without1009.png]

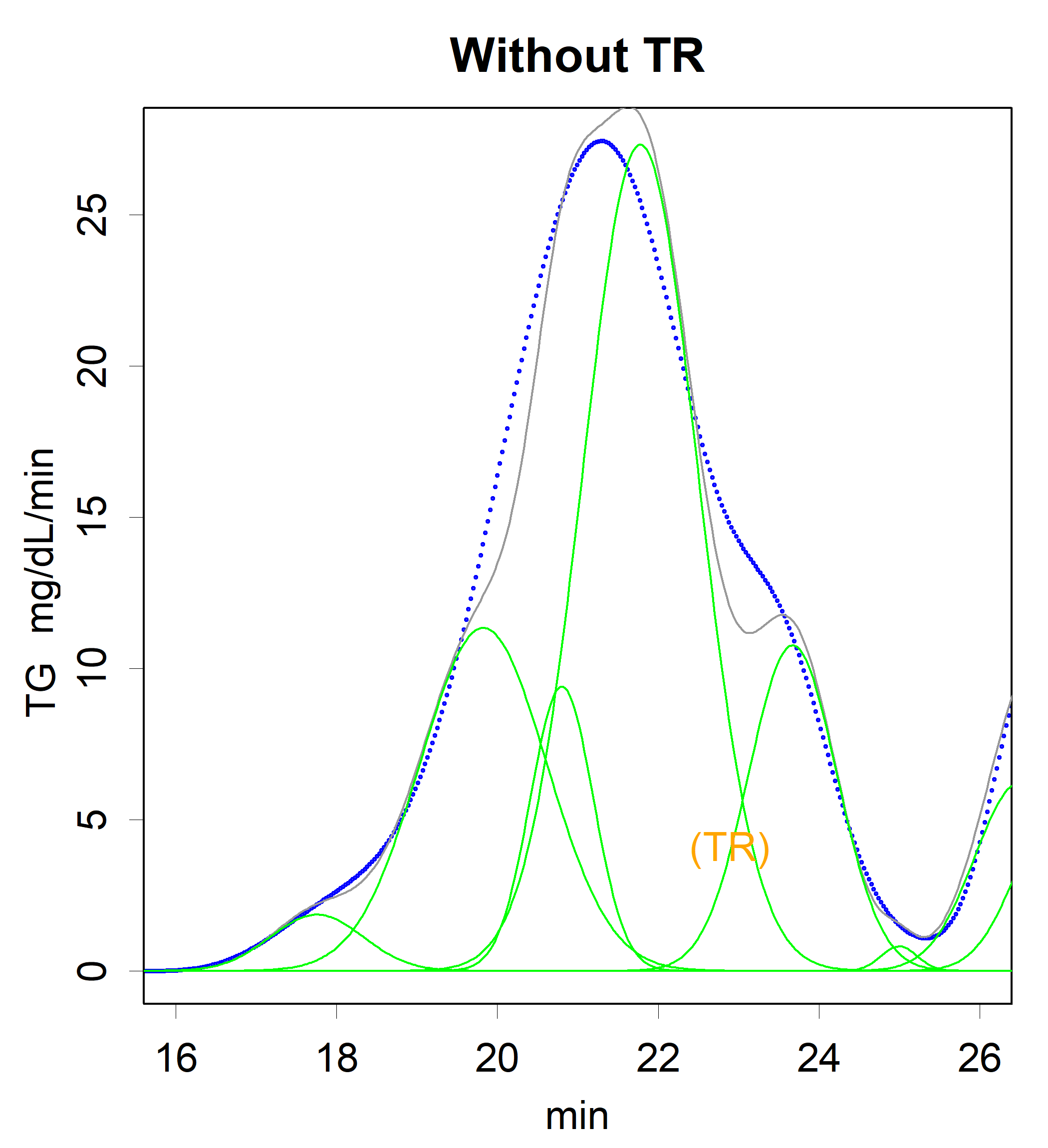

Supplement: S1 File — (ZIP) [file pone.0275066.s001.zip › supporting/fig/withoutTG/without1010.png]

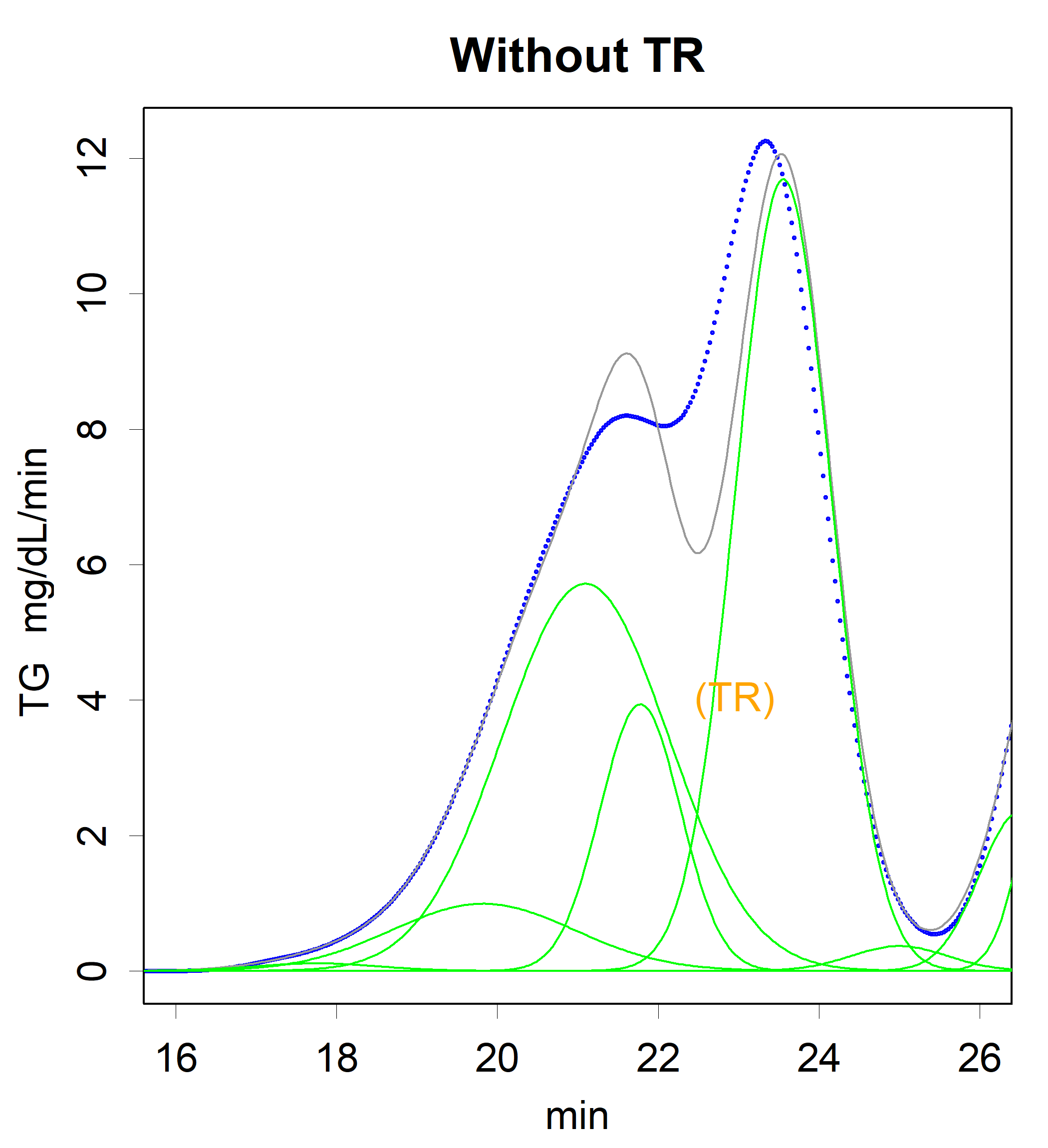

Supplement: S1 File — (ZIP) [file pone.0275066.s001.zip › supporting/fig/withoutTG/without1011.png]
