# Supplementary material for: Human lipoproteins comprise at least 12 different classes that are lognormally distributed
Source: PLoS One. 2022 Nov 10;17(11):e0275066. doi: 10.1371/journal.pone.0275066 (PMC9648703; doi:10.1371/journal.pone.0275066)
Supplement: Attachment — Submitted filename: Response to Reviewers.docx [file pone.0275066.s002.docx]

5. Review Comments to the Author

Please use the space provided to explain your answers to the questions above. You may also include additional comments for the author, including concerns about dual publication, research ethics, or publication ethics. (Please upload your review as an attachment if it exceeds 20,000 characters)

Reviewer #1: In this work, Tomokazu and colleagues provide an alternative classification of lipoprotein subclasses in human plasma after HPLC-based separation. The conclusions that are drawn by the authors, despite interesting, clash against multiple well established aspects of lipoprotein metabolism and thus should be supported by a larger and more detailed corpus of data, which is not present in the current work. In particular the manuscript lacks any mechanistical data supporting the key claims about e.g. HDL cholesterol content as well as TG-rich lipoprotein catabolism. My comments are as follows:

This is a study on the biochemical parameters of the serum in normal people. Unfortunately, metabolism, catabolism, the condition of people who are ill, and/or the use of genetically manipulated animals are beyond the scope of this study. However, as the reviewer pointed out, the impact of this article is enough to call for a rethinking of most of the studies that have been performed so far. We believe that it would be in the best interest of society to publish it soon rather than to perform additional more-comprehensive studies by ourselves.

Methods:
Line 210: “No postmeal time was specified for blood collection.” Is repeated twice

We apologize for this error. One of them was removed.

Line 211: “The age of the volunteers and the sex of the subjects were 50/50, as shown in Figure S1”. This statement is in my opinion unclear. On the one hand, there is not information regarding sex in Figure S1. What is the female to male ratio in the study? How many females/how many males? Additionally, what is meant here with “50/50”? Was the mean age in the study 50 years?

We revised this sentence. We hope this eliminates the misreading of this as the age.

Line 214: here “HDL” should be used instead than “HLD”. Also please indicate what were the “conventional methods” used to determine them. E.g. what commercial kits were used

We corrected this typo. We have also added references to two Japanese articles referred by the company that measured the samples. We do not know if the company is using the kits for their service. In any case, these are ordinary kits that can be obtained anywhere, and they will not affect the outcome of the article.

Line 216: What are the parameters used in the HPLC analysis? What column? What flow rate? What medium? Where different conditions used for analytical VS preparative HPLC? What methods were used to monitor TG and cholesterol?

As it is written in the manuscript, we asked Skylight Biotech to perform the HPLC. We do not know the parameters of the procedure or parameters and measurements of the columns, as these are their business. However, anyone can ask this company that provided that service for this information. Therefore, reproducibility, which is an obligation for an article, is ensured. For the principle of HPLC, please refer to [11] in the references. We have added this citation in the main text of the manuscript. Moreover, this is a rather common technique that is performed in many laboratories (including the laboratory in Gordon’s study). The methodology of HPLC is not the focus of this article. The difference between Gordon’s and ours is in the data analysis based on biochemical evidence.

Line 217: what do the authors mean here by hyperlipidaemic? The plasma lipid parameters of these patients as well as the type of hyperlipidaemia should be specified in the manuscript

These were patients who had been brought in with a stroke. Each of them had different abnormalities, but these data were not used in the analysis (only one person's data was used in the plot because it was easier to visualize the data this way). Therefore, there is no point in disclosing their parameters here. We have omitted this part of the description to avoid unnecessarily attracting the reader's attention.

Line 225: “class., with The amount”. Take dot away. Do not capitalize T in “The”

We fixed this typo.

Line 236-237: please either specify the MALDI-TOF-MS methods or provide a reference to a previous work where they were used.

We quoted a new reference here to introduce a description for how MALDI-TOF MS works. However, again, a commercially available service that is rather a commoditized technique was used.

Line 243: “which has better sensitivity than the biuret reaction commonly used for serum samples”. This assertion is not correct, as the BCA method is based on the biuret reaction as well. Do the authors intend that the BCA method is more sensitive compared to the Bradford method? If yes, they should change this sentence accordingly

We revised this sentence to demonstrate that BCA is based on the same bases of the biuret reaction.

Figures:
Fig 1. As far as I understand, in the figure two curves are shown that display the raw data for TG as well as for cholesterol. Both are shown in gray, which in my opinion is quite confusing, as curve-fitted data for TGs and choelesterol are then shown in red and blue. Curves for raw data should also be colored (e.g. in a different shade of red and blue), to indicate whether they refer to TG or cholesterol.

We appreciate this suggestion. We changed the colours accordingly without losing the consideration for colour-blindness. We hope this makes the figure easier to visualize.

Additionally, the SDS-PAGE gel in the left panel should be shown for clarity in a separate panel within the same figure. The SDS-PAGE panel should also indicate what are the numbers on the axes referring to (Molecular weight and elution time?)

The label "MW" has been added to the left side of the panel. However, this panel was difficult to move because its width matches the fraction number displayed directly below it. We have added this circumstance to the legend.

Last, in the SDS-PAGE the ApoB100 peak seems to reach its maximum between 10 to 19min, while no ApoB is visible after the 21 minutes fraction. Nevertheless, the authors depict a high “LDL1” peak with mean elution time of about 23 minutes. Could the authors elaborate on this? It is especially difficult to reconcile the band intensities in this silver-stained gel with the ApoB quantification data should in Fig S3, which show instead a clear ApoB100 peak around 23 minutes. A similar discrepancy between Figure 1 and S3 seems to affect the elution curve of ApoB48 as well.

The numbers are fractions, not the elution time. The visible band appeared approximately at that position on the graph (of course, the graph is continuous, whereas the fractions are discontinuous, so there would be a small difference). We apologize for the confusion. It is evident that B100 appears from VLDL to LDL1. Similarly, B48 coincides with CM. We have added explanations for this in the Result (L64) and in the figure legend.

Figure 2B: (see also comment to Fig S6). Based on this graph, the HDL fractions combined seem to contain much less cholesterol compared to the LDL fractions than expected for “normal” plasma LDL-C and HDL-C values. The authors should elaborate on this point.

We appreciate the reviewer pointing this out. This is explained in the paragraphs from L128 of the Result, and we have also added a comment to L233 of the Discussion. Additionally, the reason is evident in our newly added figure, Fig. 2C. Unfortunately, we believe the “normal” plasma LDL-C and HDL-C values are incorrect.

Fig S2: the abbreviation “TR” should be explained again in the corresponding line of the Results section (line 61) as well as in this figure’s legend. A definition is given instead only in line 80 of the results. Additionally, measurement units should be indicated on the y axes in both panels. The expression “It is unique in that it is smaller and very low in cholesterol. However, fitting is difficult without this.” Is quite unclear without having read line 80.

We appreciate the reviewer pointing this out. We arranged the figure legend and gave measurement units in the figures. We added an explanation of TR and other VLDLs to L73 and L79 of the revised Result section.

Fig S3: please see comment given for Fig 1. The authors should indicate what the two panels shown in this figure indicate. Are they curves obtained from two different samples? This should be mentioned in the legend. Additionally, what to the small gray numbers in the middle of each panel indicate?

These are the fraction numbers. We added an explanation of this in the legends.

Fig S6 The authors show a “LDL2” fraction, which nevertheless seems to appear clearly after the bulk of ApoB100 has been eluted. The HDL-cholesterol peak seems also to be much smaller than expected for an individual with an average “healthy” lipoprotein profile. Could the authors elaborate on this? How can the authors be sure that the “LDL2” peak does not in fact depict the main HDL-cholesterol peak? Additionally, what does the number “401” in the left panel indicate? In this figure as well, the SDS-PAGE gel should be shown as a separate panel, indicating what is shown in each axis.

We understand and share the impact of the fact that the HDL is considerably less than that previously thought. However, LDL2/LAC2 would be a derivative of CM (L178). Hence, it would, rather, be free from ApoB100. Additionally, the newly added figure, Fig 2C, clearly demonstrates the difference between LDL2/LAC2 and HDLs. Also, there is a limitation in the HDL volume due to the structure of ApoA-1. These are explained in the paragraphs starting from L128 using the results of some of the referenced studies and our present evidence.

We apologize about the ‘401’; it was a sample ID that we forget to erase. As the lanes of the SDS-PAGE gel are arranged to be in the same positions as those exhibited in the graph, they cannot be replaced.

Fig S8: the authors should provide correlation coefficients and p-values for linear regression for each panel.

We added these values. We have also added a figure (Fig. S6D) to show when the P-value is small. The P-values are not necessarily small here because some of the data were demonstrated as exhibiting a ‘weak relationship’ or ‘no relationship’. We have also added this information to the legend in Fig. 5 to enhance the consistency of the data.

Results:
Lines 62-63: “The attributes of the classes were determined based on the elution pattern of the major protein components (Fig. 1, S3-S6). The elution patterns of these proteins coincided with the distribution of the corresponding classes”. If the classes were assigned based on the elution pattern of the major protein components, it would then be logical that they would elute at the same time. The authors should thus delete the second statement. Also, please see my comments regarding figures 1, S3 and S6 on apparent incongruences regarding the elution times of different proteins and lipoprotein subclasses

Yes, that makes sense and is logical, but it is not always expected. Rather, this is only possible when the curve fitting was performed correctly. Therefore, this is an important result. I have added a statement explaining this.

Also, we have added explanations for Fig. 1 and S6 (new S4).

Line 63: “As the positions and scales coincided between TG and cholesterol”. The meaning of this sentence is unclear to me. Do the authors intend to say that cholesterol and TG followed a similar elution pattern within each subclass, thus suggesting a stable cholesterol-to-TG ration within that class?

No, we did not intend this. We have added explanations for the intended meaning. These parameters will only match if the particles are actually present, they are measured properly, and the curve fitting is performed reasonably well.

Line 68: “and the LDL2 fraction is devoid of B48”. Please see my comments on Figures 1, S3 and S6. Besides being devoid of ApoB48, this fractions seems not to contain ApoB100 either based on the data in Fig S3, which would clash against their classification by the authors as LDL

We have added these explanations.

Line 71: “they may supply cholesterol to the thrombus and protect it from lysis by plasmin”. This statement belongs in my opinion to the discussion section. Additionally, the authors should indicate on what this statement is based (other data? literature?)

We appreciate the reviewer for pointing this out. This should not be in the Results section, so we have moved it to the Discussion section. We also cited TK's previous article [9], which discusses the possible functions of this complex.

Lines 80-83: the authors should provide a statistical evaluation of correlation of the parameters analyzed in Fig S8, including correlation coefficients and p-values.

We have added this information.

Line 114: “HDLs were minor cholesterol carriers, contradicting the estimations of the previous study”. I find this statement problematic, and the reference to Gordon et al., (doi: 10.1021/pr100520x) who show how the ApoA-I-containing HDL fractions also carry large amounts of cholesterol in normolipidemic patients as contradicting the findings of the current study. The fact that Gordon and colleagues did not measure ApoA-I levels in each HDL fraction doesn’t cancel the fact that they nevertheless identified it as the most abundant HDL-associated protein in their MS experiments.

In identifying the peak of the HDLs, detecting Apo A-1 from a pool of several fractions was insufficient; instead, Gordon et al. had to check every fraction. However, this has been previously discussed in an article published in the previous article published in PLOS ONE [9]. Why they did this wrong is not the main focus of this article. The situation has been explained in the paragraph starting on L124, and we believe this explanation is sufficient for this article.

Rather, we will present the process that led to this conclusion by adding a new figure, Fig. 2, in the text. In the face of this new evidence, in order to disprove the evidence, ‘because it has been believed so far’ is not sufficient.

Line 118: “the structure of HDL is surrounded by ApoA-1 and therefore has a limitation in size”. The authors refer to Wu et al., doi: 10.1074/jbc.M110.209130. The authors should elaborate more on why the structural findings on this paper set a limit on HDL size based on the conformation of ApoAI.

This is also something that has already been explained; hence, we have cited the relevant reference [9] here as well. Also, this is just a side note; it is simply implying that Fig. 2C that is important as a result of this article.

Lines 123-126: these data seem in contrast, among others, with those of Gordon et al (see comment above). The authors should explain how their findings compare to other HPLC-based studies in this regard.

Unfortunately, that has been done in a previous study published in PLOS ONE [9], so it would not be beneficial to the reader if we repeated too many details. We added a citation to the previous article, and we also added explanation of this situation in L168.

Discussion:
Lines 149-152: these conclusions contradict data about lipoprotein metabolism gathered through multiple decades and should thus be supported by a much larger amount of evidence as well as a more detailed interpretation of the data. Among others, the selective modification/knockdown of key players in each lipoprotein metabolism pathway in mice as well as genetic studies in human mutation carriers have allowed to establish the current model of lipoprotein metabolism, which, despite not perfect or complete, cannot be discarded as quickly as done in this section of the current manuscript. Thus, the authors should attenuate their claims and provide them in the context of what is known e.g. in relation of the catabolism of TG-rich lipoproteins in humans.

The biochemical method used here is a direct measurement approach to the subject matter. A metabolic analysis is not a biochemical analysis; we only can assume biochemical implications from the data rather indirectly. Also, we should be aware that those studies were performed with previous methods; hence they most likely misunderstood the classes of lipoproteins (Fig. 5). We wish to be faithful to the evidence before us, not to what we believed in the past. Therefore, we are unable to follow these requests.

Science often makes mistakes. However, we do not want to discredit scientists who are doing their work with integrity because of some failure. Mistakes are inevitable, but we can always correct them later. In this case especially, we did not want to cause unnecessary anxiety to the patient or his/her physician; the knowledge is not lost now. We actually did not know it all along, so there is no need to worry. We shall find it in the future. Therefore, when we stated that, ‘Thus, the perception of lipoproteins should be updated’, we did not bother to mention in the original version of the manuscript that the scope of the update was rather catastrophic. However, we dared to mention it.

Lines 148-186: please see my previous comments about how cholesterol peaks were assigned to each subclass.

We have given a more-detailed explanation of the figures.

Reviewer #2: The manuscript describes the results of a rapid HPLC method to separate human lipoproteins. The authors identify 12 lipoprotein classes log-normally distributed. The study technically sounds and the main conclusions are supported by data. I have no major concerns but some points should be addressed.
The manuscript is hard to follow. Many data are included in the supplementary figures, this reviewer tried to download these suppl data and it was impossible to identify the different figures cited in the text.

We apologize for this. We have added a new figure, Fig. 2, which we believe is particularly important in proving the content. Also, we reduced the number of figures in the supplement. We hope these have improved the readability.

The levels of VLDL, Lp(a), and TR (triglyceride-rich lipoproteins) showed no relationship, suggesting that they were produced independently (Fig. S8AB). I cannot download this figure, but it is difficult to believe that the levels of VLDL and TR were produced independently. How? VLDL is one of the main determinants of TR.

This is unfortunate. It is true that it would have been difficult to understand without the supplements. However, the data actually demonstrated this phenomenon. We added an explanation for this in the discussion (L168). It is possible that VLDL, Lp(a), and TR play different roles in supplying cholesterol and TG.

Define mHDL (mature HDL?)

We added an explanation for this (L119).

Since there is significant overlap in several lipoprotein classes, TG and cholesterol distribution for these lipoproteins is a critical point. This point should be clearly addressed.

We appreciate the reviewer pointing this out. As we looked at more patient data after this study, we observed that it is sometimes difficult to accurately separate some of the classes. We explained this situation in the Result (L70) and Discussion (L200). However, the measurements were within a certain range, so it is assumed that the error was not so large here for the healthy samples (Fig. 3).

6. PLOS authors have the option to publish the peer review history of their article ([what does this mean?](https://journals.plos.org/plosone/s/editorial-and-peer-review-process#loc-peer-review-history)). If published, this will include your full peer review and any attached files.


If you choose “no”, your identity will remain anonymous but your review may still be made public.


**Do you want your identity to be public for this peer review?** For information about this choice, including consent withdrawal, please see our [Privacy Policy](https://www.plos.org/privacy-policy).

Reviewer #1: No

Reviewer #2: No

[NOTE: If reviewer comments were submitted as an attachment file, they will be attached to this email and accessible via the submission site. Please log into your account, locate the manuscript record, and check for the action link "View Attachments". If this link does not appear, there are no attachment files.]

While revising your submission, please upload your figure files to the Preflight Analysis and Conversion Engine (PACE) digital diagnostic tool, <https://pacev2.apexcovantage.com/>. PACE helps ensure that figures meet PLOS requirements. To use PACE, you must first register as a user. Registration is free. Then, login and navigate to the UPLOAD tab, where you will find detailed instructions on how to use the tool. If you encounter any issues or have any questions when using PACE, please email PLOS at [figures@plos.org](mailto:figures@plos.org). Please note that Supporting Information files do not need this step.

*In compliance with data protection regulations, you may request that we remove your personal registration details at any time.* [*(Remove my information/details)*](https://www.editorialmanager.com/pone/login.asp?a=r)*. Please contact the publication office if you have any questions.*
